# Aryl hydrocarbon receptor: a potential target for natural products in the treatment of inflammatory bowel disease

**DOI:** 10.3389/fimmu.2026.1720700

**Published:** 2026-02-04

**Authors:** Lv Ran, Chunrun Li, Peng Wang, Junmei Tang, Zhengwu Qu, Yanwei Hao, Yi Zhang

**Affiliations:** 1 Hospital of Chengdu University of Traditional Chinese Medicine, Chengdu, Sichuan, China; 2 Clinical Medical College, Chengdu University of Traditional Chinese Medicine, Chengdu, Sichuan, China

**Keywords:** Aryl Hydrocarbon Receptor (AHR), inflammatory bowel disease (IBD), natural products, intestinal barrier, gut microbiota, immune modulation

## Abstract

Inflammatory bowel disease (IBD) is a chronic intestinal inflammatory disease driven by genetic, immune, and environmental factors, and its incidence continues to increase worldwide. The existing therapies often face the limitations of insufficient response, obvious side effects, and high medical burden, so it is urgent to develop safe and effective intervention strategies based on new targets. The aryl hydrocarbon receptor (AHR), a crucial environmental sensor, plays an essential role in preserving intestinal barrier function, modulating immune homeostasis, and facilitating microbiota-host interactions through the integration of ligand-mediated signals. Notably, natural products constitute a major source of AHR ligands and exhibit multiple therapeutic potentials to repair the intestinal barrier, modulate immunity, and remodel the microbiota through targeted activation of AHR. This provides a unique theoretical perspective for developing innovative therapeutic strategies. In this review, we systematically explore the fundamental relationship between AHR and IBD, and introduce the receptor's biological characteristics and regulatory mechanisms in detail. In addition, this article further emphasizes the pharmacological properties and molecular mechanisms of various natural products that target AHR as prospective treatments and evaluates their potential for clinical applications.

## 1 Introduction

Inflammatory bowel disease (IBD), primarily comprising Crohn's disease (CD) and Ulcerative colitis (UC), is a worldwide disease characterized by chronic and relapsing intestinal inflammation. While the incidence of IBD has plateaued in developed nations over the past three decades, a significant increase has been observed in newly industrialized regions such as China and India (1). Currently, IBD afflicts more than 6.8 million people worldwide, with a notable trend toward younger age. This shift not only seriously impairs patients' quality of life but also imposes a substantial socioeconomic burden. Although the exact etiology of IBD remains incompletely elucidated, it is widely believed to arise from a complex interaction of genetic susceptibility, aberrant host immune responses, impaired mucosal barrier function, and gut microbial dysbiosis (2). Current therapeutic strategies predominantly involve corticosteroids, immunosuppressants, and biologic agents. However, long-term use of these therapies is associated with increased risks of infection, metabolic disorders, and considerable healthcare costs, highlighting the imperative for novel therapeutic targets.

The aryl hydrocarbon receptor (AHR) is a pivotal ligand-activated sensor and transcription factor that is ubiquitously expressed in a wide variety of cells. Research has demonstrated that AHR has tissue-specific regulatory effects, particularly in immune cells such as T cells and macrophages, as well as epithelial cells of barrier tissues, including the skin and gut (3). Initially, AHR was recognized for its involvement in mediating the detoxification of environmental toxins. However, recent investigations have expanded its recognized functions to include immune regulation, tissue repair, and the maintenance of intestinal microbial homeostasis (4). Notably, environmental factors such as dietary components, air pollutants, and severe imbalance of gut microbiota contribute to the onset and progression of IBD. As a ligand-activated transcription factor, AHR serves as a central molecular integrator connecting environmental signals to the maintenance of intestinal immune equilibrium. Its ability to detect endogenous and exogenous ligands is essential for regulating epithelial barrier restoration, immune cell differentiation, and inflammatory signaling pathways. Consequently, the role of AHR as an environmental sensor constitutes a critical element in the pathogenesis of IBD.

Natural products are characterized as organic small molecules originating from secondary metabolic pathways within living organisms. These compounds are distinguished by their extensive structural diversity and significant bioactivity. The plant-derived compounds discussed in this review mainly include phenolic compounds, which are abundant in plants and possess antioxidant properties; alkaloids, which are recognized for their powerful physiological effects; structurally diverse terpenoids and steroids; and glycosides, which are composed of glycones and aglycones. Given their chemical heterogeneity and potential to act as high-affinity ligands for AHR, natural products represent an optimal resource for investigating AHR modulators. This article focuses on the complex regulatory roles of AHR in the pathogenesis and progression of IBD. Furthermore, the review explores the effects of natural products targeting AHR in alleviating IBD, alongside elucidating their underlying pharmacological mechanisms. This study aims to furnish scientific evidence supporting the development of targeted, AHR-based precision therapeutic strategies and pharmacological agents for IBD.

## 2 Structure and signaling pathways of AHR

### 2.1 Molecular structure of AHR

**Figure 1:**
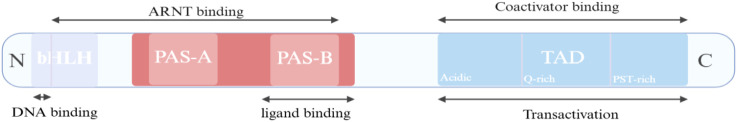
Schematic diagram of the AHR protein domains. AHR contains three main domains: the bHLH domain, the PAS domain, and the TAD. The N-terminal bHLH domain facilitates interactions with the chaperones HSP90 and XAP2, promotes ARNT dimerization, and enables DNA binding. This is followed by two PAS domains: PAS-A contributes to stabilizing the ARNT dimer structure, while the PAS-B domain serves as the primary ligand-binding region. The C-terminal region is the transactivation domain, which is involved in the recruitment of coactivators and transactivation. It consists of three subdomains: an acidic residue-rich subdomain, a glutamic acid-rich subdomain, and a proline/serine/threonine-rich domain.

The AHR is a highly conserved, ligand-activated transcription factor that belongs to the basic helix-loop-helix-per-aryl-hydrocarbon receptor nuclear translocator-sim (bHLH-PAS) superfamily. This protein, consisting of 848 amino acids, contains several functionally synergistic domains, including an N-terminal bHLH domain, two Per-Arnt-Sim (PAS) domains designated PAS-A and PAS-B, and a C-terminal transcriptional activation domain (TAD) (5). The bHLH domain facilitates AHR binding to the xenobiotic response element (XRE/DRE; consensus sequence *5′-TGCGTG-3′*) and promotes heterodimerization with the aryl hydrocarbon receptor nuclear translocator (ARNT). The PAS-A domain plays a critical role in stabilizing the formation of the AHR-ARNT heterodimer. Conversely, the PAS-B domain has been found to harbor a hydrophobic ligand-binding pocket, which serves as the principal site for ligand recognition and the initiation of receptor activation (6). The C-terminal TAD recruits transcriptional co-regulators through its glutamine-rich region and clusters of acidic amino acids, thereby facilitating the expression of target genes. Notably, the TAD exhibits considerable structural variability relative to the conserved bHLH-PAS core, a feature that is closely associated with its functional heterogeneity in transcriptional regulation (7). (**Figure 1**).

### 2.2 AHR ligand diversity

The activity of the AHR is tightly regulated through its ligand binding. It has been established that ligand interaction with the hydrophobic pocket of the PAS-B domain induces conformational changes in the receptor, which subsequently promote its nuclear translocation and transcriptional activation (8). A variety of AHR ligands have been identified, which can be classified into two main categories based on their origin: exogenous and endogenous (9). Exogenous ligands primarily encompass environmental pollutants and dietary components. Industrial contaminants, including halogenated aromatic hydrocarbons, polycyclic aromatic hydrocarbons, and polychlorinated biphenyls, have been shown to typically exhibit high affinity for AHR and robustly activate the receptor. A prototypical example is 2,3,7,8-tetrachlorodibenzo-p-dioxin (TCDD), which activates *CYP1A1*, *CYP1A2*, and *CYP1B1* via AHR, facilitating the metabolic conversion of procarcinogens into genotoxic epoxides and thereby contributing to inflammation and carcinogenesis (10). Conversely, natural dietary molecules generally function as low-affinity ligands, often termed “pro-ligand” (11), which require biochemical conversion to become active. These compounds lack direct binding capability and necessitate specific microenvironmental conditions, such as exposure to gastric acid or reactive oxygen species, to undergo chemical transformation into functional ligands. Endogenous ligands primarily originate from the host's intrinsic metabolic processes, as well as from metabolites and derivatives produced through a collaborative microbiota-host metabolism of tryptophan (Trp) (12). Within the host metabolic pathway, more than 95% of Trp is catalyzed into kynurenine (Kyn) by the enzymes tryptophan 2,3-dioxygenase (*TDO*) or indoleamine 2,3-dioxygenase (*IDO*). Kyn then undergoes non-enzymatic reactions, including spontaneous oxidation or photo-oxidation, resulting in the formation of tricyclic aromatic condensation products, such as 6-formylindolo[3,2-b]carbazole (FICZ), which function as potent agonists (13). Regarding microbial metabolism, bacterial tryptophanase converts Trp into indole (14), which is further metabolized into various AHR agonists, including indole-3-acetic acid (IAA), tryptamine, and indole-3-aldehyde (IAld). These microbial metabolites have been shown to exert anti-inflammatory effects by activating the AHR and modulating the intestinal immune microenvironment, a crucial mechanism for maintaining mucosal barrier homeostasis in the host (15). Additionally, the Trp and serotonin metabolic pathways can independently generate Trp-derived ligands outside the *IDO/TDO* pathway (16). (**Table 1**) It is important to clarify the terminology used in this review regarding AHR-interacting molecules. The ligands summarized in **Table 1** are supported by direct binding assays or are widely recognized as such in the literature (17). In contrast, many natural products discussed in Section 5 are characterized as AHR activators, agonists, or modulators based on functional assays (e.g., induction of *CYP1A1*, nuclear translocation). Their direct binding affinity to AHR awaits further validation using standardized biochemical approaches.

**Table 1:** AHR ligands.

Ligands	Category	Origin
Dioxins and dioxin-like		
2,3,7,8-Tetrachlorodibenzo-p-dioxin(TCDD)	Exogenous	Environmental pollutants
2,3,4,7,8-Pentachlorodibenzofuran (PeCDF)	Exogenous	Environmental pollutants
3-Methylcholanthrene (3-MC)	Exogenous	Environmental pollutants
Benzo[b]fluoranthene	Exogenous	Environmental pollutants
Polycyclic aromatic hydrocarbons (PAHs)	Exogenous	Tobacco smoke and environmental pollutants
Naphthalene	Exogenous	Tobacco smoke and industrial emissions
Polychlorinated biphenyls (PCBs)	Exogenous	Industrial chemicals
Hexachlorobenzene (HCB)	Exogenous	Fungicide and industrial chemical
β-Naphthoflavone (BNF)	Exogenous	Synthetic compound
Tryptophan (Trp) metabolites		
Kynurenine (Kyn)	Endogenous	Host metabolism
Kynurenic acid (KA)	Endogenous	Host metabolism
Trace-extended aromatic condensation products (TEACOPs)	Endogenous	Host metabolism
Indole-3-pyruvate (I3P)	Endogenous	Host metabolism
Indole-3-pyruvic acid (I3PA)	Endogenous	Host metabolism
6-Formylindolo[3,2-b]carbazole (FICZ)	Endogenous	Photo-oxidation
Indole-3-acetic acid (IAA)	Endogenous	Microbiota metabolism
Indole-3-aldehyde (I3A)	Endogenous	Microbiota metabolism
Indole-3-acetaldehyde (IAAId)	Endogenous	Microbiota metabolism
Tryptamine (TA)	Endogenous	Microbiota metabolism
Indoxyl-3-sulfate (I3S)	Endogenous	Microbiota metabolism and host metabolism
Indole metabolites		
Indole metabolites	Endogenous	Dietary metabolite
Indolo[3,3-b]carbazole	Endogenous	Dietary metabolite
2-(Indol-3-ylmethyl)-3,30-diindolylmethane (Ltr-1)	Endogenous	Dietary metabolite
3,30-Diindolylmethane (DIM)	Endogenous	Dietary metabolite
2-(1'*H*-indole-30-carbonyl)-thiazole-4-carboxylic acid methyl ester (ITE)	Endogenous	Host metabolism
Dietary		
Quercetin (flavonoid)	Exogenous	Fruits, vegetables, and plants
Kaempferol (flavonoid)	Exogenous	Fruits, vegetables, and plants
Sulfophane	Exogenous	Cruciferous vegetables
Indole-3-carbinol (I3C)	Exogenous	Cruciferous vegetables
5,11-Diihydroindolo-[3,2-b]carbazole (ICZ)	Exogenous	Cruciferous vegetables
3,3'-Diindolymethane (DIM)	Exogenous	Cruciferous vegetables
Docosahexaenoic acid (DHA)	Exogenous	ω-3 fatty acids derived from fatty fish
Eicosapentaenoic acid (EPA)	Exogenous	ω-3 fatty acids derived from fatty fish
3,5,4'-Trihydroxystilbene (resveratrol)	Exogenous	Fruits, vegetables, and plants
Inulin	Exogenous	Dietary fibres
Others		
Indigorubin	Exogenous	Phytochemical
Indigo	Exogenous	Phytochemical
Bilirubin	Endogenous	Haem-derived metabolism
Biliverdin	Endogenous	Haem-derived metabolism
Lipoxin4A	Endogenous	Arachidonic acid metabolites
Prostaglandin G_2_ (PGG_2_)	Endogenous	Arachidonic acid metabolites
Prostaglandin E_2_ (PGE_2_)	Endogenous	Arachidonic acid metabolites
Leukotriene B_4_ (LTB_4_)	Endogenous	Arachidonic acid metabolites
Hydroxyeicosatrienoic acid (12R-HETE)	Endogenous	Arachidonic acid metabolites

The diversity of AHR ligands leads to distinct interactions with receptor binding domains, including ligand-specific conformational modifications, variations in nuclear retention time, and differential downstream signaling outcomes. Environmental pollutants with a high affinity for AHR, such as TCDD, exhibit resistance to degradation by *CYP1* enzymes, resulting in sustained receptor activation that contributes to metabolic disorders, pro-inflammatory responses, and an increased carcinogenic risk (13, 18). In contrast, AHR ligands originating from the microbiome or dietary sources have demonstrated protective effects by maintaining mucosal homeostasis through controlled biotransformation and rapid metabolic clearance. At appropriate concentrations, these ligands possess therapeutic potential, encompassing anti-inflammatory, immunomodulatory, and barrier-protective properties (9).

### 2.3 The AHR signaling pathway

**Figure 2:**
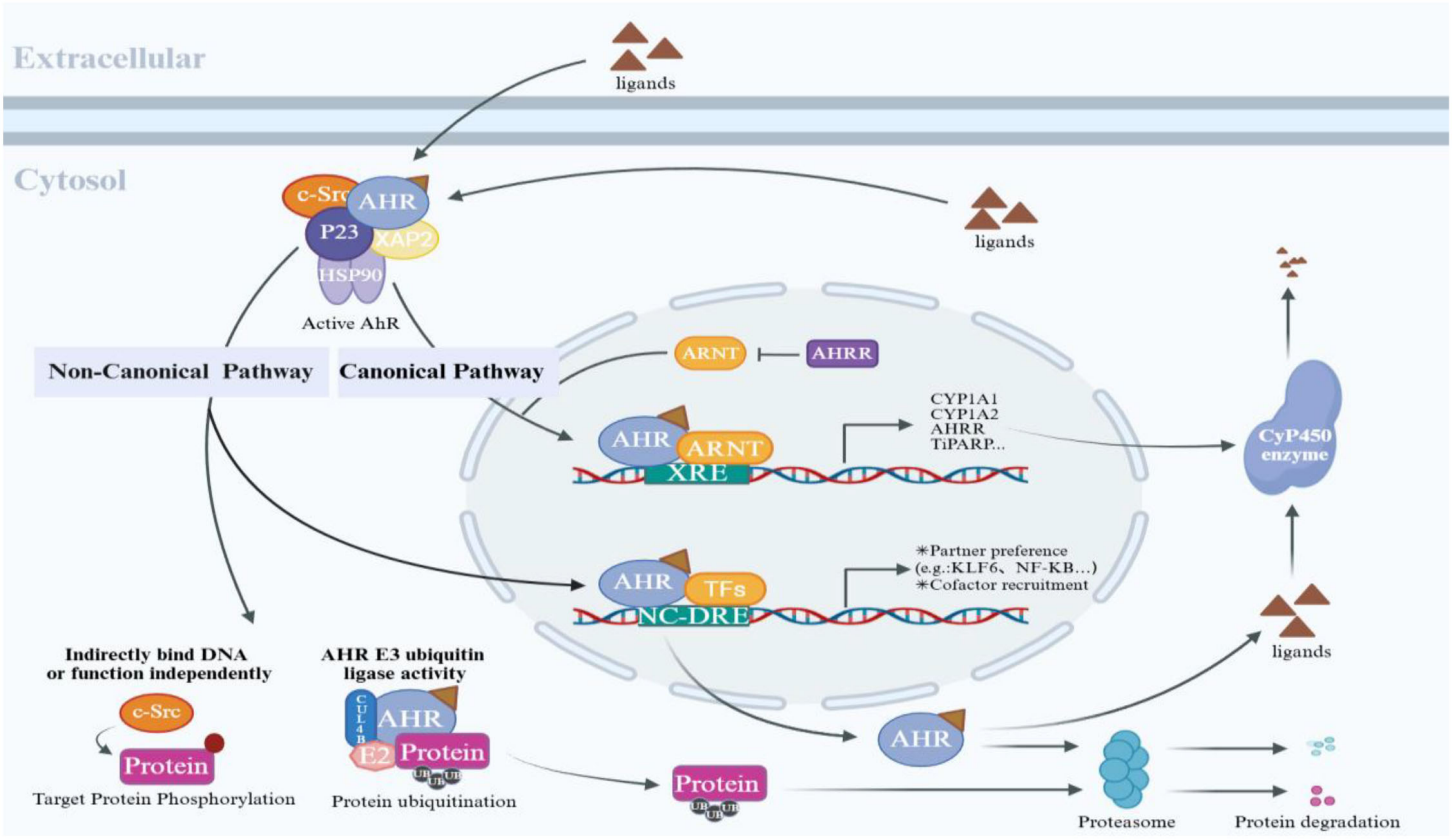
AHR-mediated canonical and non-canonical signaling pathways: The inactive AHR resides in the cytoplasm with molecular chaperone proteins (HSP90, XAP2, p23, c-Src). Ligand binding triggers complex dissociation and induces AHR nuclear translocation. In the canonical pathway, nuclear AHR dimerizes with ARNT and binds to XREs, initiating transcription of target genes, including cytochrome P450 enzymes (*CYP1A1*/CYP1A2), *AHRR*, and *TiPARP*. *AHRR* blocks AHR-ARNT complex formation by competitively binding to ARNT, while *TiPARP* promotes AHR ubiquitination through PARsylation modification, ultimately leading to proteasomal degradation, together forming a negative feedback loop. The non-canonical pathway encompasses both genomic and non-genomic regulation. The former activates non-XRE-dependent genes through interactions between AHR and transcription factors such as *KLF6* and NF-κB. The latter forms the AHR-CUL4B complex, which exerts an E3 ubiquitin ligase function (mediating the degradation of substrates, including steroid receptors) and triggers a phosphorylation cascade via ligand-released c-Src.

The activation of the AHR signaling pathway involves a series of molecular steps, including ligand binding, conformational rearrangement, nuclear translocation, and transcriptional regulation. Under basal conditions, cytosolic AHR exists in an inactive multiprotein complex alongside heat shock protein 90, the AHR-interacting protein, and the p23 chaperone (19). Ligand engagement at the PAS-B domain induces conformational rearrangements within the AHR complex, thereby exposing the nuclear localization signal located in the bHLH domain, as well as the adjacent nuclear transport signal. This process facilitates recognition by importin-β, thereby promoting the subsequent nuclear translocation of AHR (20). Once inside the nucleus, AHR heterodimerizes with ARNT, enabling the complex to bind the dioxin response element (DRE) within the promoters of target genes and initiate their transcription. The canonical AHR signaling pathway predominantly governs the transcriptional activation of cytochrome P450 enzymes, specifically *CYP1A1*, CYP1A2, and *CYP1B1*. These enzymes are integral to the metabolism of xenobiotic compounds and are essential for mediating adaptive responses and toxicity induced by exogenous ligands (21). Additionally, AHR signaling has been implicated in immune regulation through modulation of the AHR repressor (*AHRR*) and interleukin-22 (IL-22) expression (22). Beyond the canonical pathway, AHR engages in non-canonical activation mechanisms via cross-talk with multiple signaling cascades, including nuclear factor-κB (NF-κB), nuclear factor erythroid 2-related factor 2 (Nrf2), epidermal growth factor receptor, steroid hormone receptors, and the Janus kinase-signal transducer and activator of transcription pathway, collectively influencing gene expression (23). (**Figure 2**) For example, AHR can directly interact with *RelA* and *RelB* of the NF-κB complex, interfering with their transcriptional programs, thereby suppressing or altering the expression of NF-κB-driven pro-inflammatory genes. One study demonstrated that the AHR agonist β-naphthoflavone suppresses astrocytic differentiation in glioma cells by inhibiting IL-6 expression and downstream *STAT3* activation (24). This suggests that AHR modulates JAK/STAT pathway activity and cellular phenotypes through the regulation of cytokines and related signaling molecules. Notably, the biological effects mediated by *STAT3* are context-dependent: in cells such as ILC3, *STAT3* cooperates with IL-22 signaling to exert anti-inflammatory effects, whereas in other immune cells, *STAT3* interaction with the NF-κB pathway may drive pro-inflammatory responses. Therefore, elucidating the dual role of AHR in these distinct *STAT3*-related pathways is essential for understanding its complex immunoregulatory functions. AHR has also been shown to regulate the expression of several cytokines that activate the JAK/STAT pathway, including IL-2, IL-10, IL-21, and IL-22. However, experimental evidence delineating the interactions between AHR and these signaling pathways remains limited and requires further investigation (23). Notably, the AHR pathway functions as a versatile sensing and transcriptional hub, coordinating metabolic adaptation in response to exogenous stimuli. It plays critical roles in various physiological and pathological processes, such as immune homeostasis, inflammation, epithelial barrier integrity, cell differentiation, and tissue maintenance-mediated through both canonical XRE-dependent mechanisms and non-canonical signaling pathways (17).

## 3 Inhibition of the AHR pathway and its association with IBD

The inhibition of the AHR signaling pathway is critically implicated in the pathogenesis of IBD, as supported by extensive research evidence. Genome-wide association studies have confirmed significant correlations between single-nucleotide polymorphisms within the AHR gene locus and an increased susceptibility to IBD (25). These genetic variations have been shown to impair the binding affinity of endogenous ligands to AHR by inducing conformation changes in its ligand-binding domain. Clinical cohort analyses reveal that both AHR mRNA and protein expression levels in the colonic mucosa of IBD patients are markedly reduced compared to healthy controls, with these reductions exhibiting a negative association with disease activity indices (26). Additionally, Metabolomic investigations indicate diminished concentrations of AHR ligands, particularly Trp-derived metabolites, in the fecal matter of IBD patients, concomitant with decreased *IDO1* enzymatic activity. This biochemical milieu contributes to persistent inhibition of the AHR signaling pathway (27).

The current investigation provides direct evidence supporting the protective function of AHR through the use of animal models. In comparison to wild-type mice, AHR-knockout mice exhibited exacerbated intestinal epithelial injury, increased barrier permeability, and elevated levels of pro-inflammatory cytokines, specifically interleukin-6 (IL-6) and tumor necrosis factor-alpha (TNF-α), in dextran sulfate sodium (DSS)-induced colitis models (28). Notably, dietary supplementation with I3C was found to ameliorate these pathological phenotypes, enhance the expression of tight junction proteins (TJs) such as Occludin and Claudin-1, and restore intestinal barrier integrity (29). Furthermore, fecal microbiota transplantation (FMT) experiments have elucidated the mechanistic interplay between the gut microbiota and AHR signaling. FMT was shown to reestablish microbiota homeostasis in DSS-induced colitis mice, concomitantly increasing colonic AHR expression and IAA levels. Such activation facilitated AHR-dependent upregulation of anti-inflammatory cytokines IL-10 and TGF-β, thereby indicating that microbiota-derived metabolites regulate intestinal immune equilibrium via the AHR pathway (30). Investigations *in vitro* further corroborate the regulatory role of AHR. Specifically, treatment of Caco-2 cells, which serve as a model of intestinal epithelium, with the AHR agonist FICZ has been identified to enhance barrier integrity and counteract the increase in epithelial permeability induced by TNF-α and IFN-γ (31). In summary, evidence from multiple levels consistently suggests that inhibition of the AHR pathway constitutes an important factor in the pathogenesis of IBD. This includes aspects such as genetic predisposition, reduced receptor expression, ligand deficiency, as well as findings from both *ex vivo* and *in vivo* functional studies. The underlying mechanisms implicated involve disruption of the intestinal barrier, dysregulation of immune homeostasis, and altered interactions between the microbiota and the host.

## 4 Activation of the AHR pathway protects against IBD

### 4.1 Regulation of the intestinal mucosal barrier

The intestinal mucosal barrier is a highly selective and permeable system, primarily responsible for modulating the absorption of water, electrolytes, and nutrients. Additionally, it serves as a crucial physical and immunological defense mechanism against pathogens and deleterious antigens within the intestinal lumen, while also facilitating interactions between the commensal microbiota and the host immune system (32). The structural integrity of the intestinal tract relies on several components, including a biological barrier composed of commensal microbiota; a physical barrier, which encompasses the mucin bilayer, mucus, and TJs of intestinal epithelial cells (IECs); a chemical barrier, consisting of antimicrobial peptides and sIgA; and an immune barrier, comprising gut-associated lymphoid tissue and various immune cells. Together, these elements ensure selective permeability, protection against pathogens, and the maintenance of immune homeostasis (33). Dysfunction of this barrier is recognized as a pivotal pathogenic factor in IBD. The AHR contributes to the preservation of barrier integrity through multidimensional mechanisms, with deficits in its function directly promoting IBD progression. It is well acknowledged that AHR's regulation of the biological and immune barriers involves intricate microbiota-host interactions and complex immune signaling networks, which will be discussed in detail in subsequent sections. The present section is devoted to elucidating the mechanisms by which AHR regulates the physical and chemical components of the intestinal mucosal barrier.

#### 4.1.1 Physical barrier

IECs constitute the fundamental structural component of the intestinal physical barrier, functioning synergistically with TJs and the bacterial biofilm to preserve barrier integrity. The AHR, which is abundantly expressed in IECs, has been demonstrated to significantly enhance barrier protection upon activation by endogenous ligands (11). As the primary interface for exogenous AHR ligands, IECs mediate AHR activation, which subsequently induces the expression of metabolic enzymes such as *CYP1A1* and *CYP1B1*. These enzymes promote ligand clearance and contribute to the maintenance of systemic ligand balance. Importantly, persistent expression of *CYP1A1* can lead to excessive ligand metabolism, thereby mimicking AHR functional deficiencies. However, supplementation with the ligand precursor I3C has been shown to counteract this effect, underscoring the role of IECs as key “gatekeepers” in regulating systemic AHR ligand availability through metabolic control (34). Furthermore, AHR activation has been found to promote the degradation of Wnt receptors and inhibit hyperactivation of the Wnt/β-catenin signaling pathway via the direct transcriptional upregulation of the E3 ubiquitin ligases *RNF43* and *ZNRF3* (35). This regulatory mechanism is essential for maintaining the proliferation-differentiation equilibrium of intestinal stem cells (ISCs) and safeguarding the regenerative capacity of the intestinal barrier. TJs, comprising proteins such as tight junction protein-1 (ZO-1), Occludin, and claudins, represent another core component of the physical barrier. These structures regulate paracellular permeability, facilitate the selective transport of materials, and maintain cellular polarity. Disruption of TJs by pathogenic agents has been implicated in barrier dysfunction, a key factor in the pathogenesis of IBD (36). Experimental evidence suggests that the AHR agonist FICZ protects the intestinal barrier by restoring the expression and cellular localization of TJs, as demonstrated in mice with DSS-induced colitis and in TNF-α/IFN-γ-treated Caco-2 cell monolayers (31). Similarly, UroA and its analogue UAS03 have been reported to significantly decrease intestinal permeability and alleviate colitis symptoms by upregulating expressions of Claudin-4, ZO-1, and Occludin-1 through the AHR-Nrf2 pathway (37). Conversely, the traditional herbal formulation *Scutellaria baicalensis* Tang activates AHR by regulating Trp metabolism, inhibits the MLCK/p-MLC signaling cascade, and enhances TJ protein expression at both transcriptional and translational levels, thereby facilitating barrier repair in UC models (38).

#### 4.1.2 Chemical barrier

Beyond its role in maintaining the physical barrier, the AHR is also integral to the regulation of the chemical barrier within the intestinal mucosa. Mucins, which constitute a critical component of the intestinal mucosal chemical barrier, function to prevent bacterial adherence to the intestinal epithelium and promote the entrapment and clearance of pathogens via intestinal peristalsis (39). Research has shown that IAA upregulates the expression of key molecules involved in mucin sulfation, including 3'-phosphoadenosine 5'-phosphosulfate synthase 2 (Papss2) and solute carrier family 35 member B3 (Slc35b3), through activation of the AHR signaling pathway. This modulation contributes to mucin sulfation processes and fortifies intestinal barrier integrity (40). Furthermore, activation of AHR by FICZ has been shown to upregulate Muc2 expression and increase the number of cuprocytes, thereby reducing bacterial translocation and ameliorating DSS-induced colitis (41). Similarly, Uro A promotes MUC2 protein synthesis and mucus secretion via the AHR-Nrf2 pathway (42). In terms of antimicrobial defense, alpha-defensin 1 (Defα1), secreted by Paneth cells, represents a pivotal antimicrobial peptide (AMP) essential for maintaining intestinal homeostasis. Reduced expression of AHR and Defα1 has been observed in IECs from ileal tissues of patients with CD as well as in murine models of colitis. Mechanistically, AHR directly induces Defα1 expression by binding to DRE, and impairments in this pathway lead to microbiota dysbiosis and exacerbation of colitis (43). Furthermore, whole-transcriptome analyses suggest that AHR may broadly regulate the expression of multiple AMPs, although the specific mechanism requires further exploration. A recent study also finds that AHR activation promotes the production and secretion of β-defensin 1 (BD-1) by columnar epithelial cells, thereby inhibiting microbiota imbalance and alleviating symptoms of colitis (44).

### 4.2 Regulation of intestinal immunity homeostasis

The occurrence and development of IBD are closely associated with dysregulated activation of immune cells and disruption of the cytokine network. Within this complex immunopathological process, AHR serves as a critical intersection between environmental signals and the immune system, playing an important role in modulating the balance of immune cell subsets and reshaping the cytokine network. The AHR not only governs the development, proliferation, and functional differentiation of intestinal immune cells but also precisely regulates the expression and signaling of key cytokines such as IL-22, IL-10, and TNF-α. Consequently, it significantly influences intestinal mucosal barrier integrity and immune responses, underscoring its great significance for maintaining intestinal immune homeostasis (45).

#### 4.2.1 Regulating the balance of immune cell subsets

Innate lymphoid cells (ILCs) serve as terminal effector cells within barrier tissues, responding to tissue injury by differentiating into distinct subsets, including ILC1, ILC2, and ILC3, and are crucial for maintaining intestinal immune homeostasis. Among these, the ILC3 subset, particularly the ILC22 population, secretes IL-22 via an AHR-dependent pathway. This secretion promotes the proliferation, survival, and antimicrobial peptide production of IECs, thereby enhancing the mucosal barrier’s resistance to infection (46). AHR is an important regulator of IL-22 produced by ILC3. Its absence leads to a marked decrease in NCR^+^ILC3 within the intestinal lamina propria and a concomitant reduction of IL-22 levels, ultimately impairing mucosal repair processes (47). Abnormal AHR signaling in patients with IBD is closely linked to an imbalance in ILCs subpopulations. In CD patients, there is an increased ratio of ILC1 to ILC2 in the intestinal milieu, accompanied by a decrease in NCR^+^ ILC3, IL-22 deficiency, and elevated pro-inflammatory factors such as IFN-γ and IL-17 (48, 49). Conversely, UC is characterized by expansion of ILC2 and loss of NCR^+^ ILC3, which correlates with increased epithelial permeability (50). Mechanistically, reduced AHR activity downregulates the expression of the transcription factor *RORγt* in ILC3, driving their conversion into a pro-inflammatory ILC1 phenotype and exacerbating the Th1-mediated inflammatory responses (51). Concurrently, AHR suppresses the expression of the IL-33 receptor on ILC2 by inhibiting the transcription factor *Gfi1*, thereby reducing the secretion of IL-5 and IL-13 (52). AHR deficiency leads to the aberrant amplification of ILC2, which in turn promotes eosinophil infiltration and the initiation of type 2 immune responses. Collectively, these findings highlight the essential role of AHR in regulating intestinal immunity in IBD through the maintenance of ILC subset equilibrium.

Helper T cells contain a variety of subsets with different functions and molecular phenotypes. Among them, Th17 cells, regulatory T cells (Treg cells), and type 1 regulatory T cells (Tr1) exhibit relatively high expression levels of AHR (53). AHR not only indirectly regulates T cell responses by modulating antigen-presenting cells but also directly participates in signaling within T cells, thereby significantly influencing their polarization and functional fate. Th17 cells participate in the protection of the intestinal barrier primarily through the secretion of IL-22. However, these cells are abnormally activated in IBD, resulting in excessive production of IL-17, which promotes inflammation. Conversely, Treg cells sustain intestinal immune tolerance by mediating immunosuppression via the release of TGF-β and IL-10. Clinical studies have demonstrated an increased infiltration of Th17 cells alongside a reduction in Treg cell populations within the intestine in IBD patients. This imbalance in the Th17/Treg ratio is directly implicated in the exacerbation of inflammatory processes, underscoring the important role of Th17/Treg immune dysregulation in IBD pathogenesis (54). As a key regulator of Th17 and Treg cells differentiation, AHR regulates their balance through mechanisms contingent upon the microenvironment. *In vivo* studies reveal that AHR signaling suppresses Th17 differentiation, as evidenced by a significant elevation of intestinal Th17 cells in AHR-knockout mice. Furthermore, the AHR ligand TCDD has been shown to inhibit IL-17 promoter activity via DNA methylation, thereby attenuating colitis (55). Contrastingly, *in vitro* data suggest that AHR may facilitate Th17 differentiation by promoting ubiquitination and subsequent degradation of STAT1, indicating that AHR’s effects are influenced by the surrounding microenvironment (56). Regarding Treg cells, AHR enhances their functionality through multiple pathways: activation of AHR upregulates the expression of *Foxp3*, stimulates the production of the retinoic acid-metabolizing enzyme *ALDH1A1* (57), and induces expression of the gut-homing receptor *Gpr15* (58). These actions collectively preserve both the number and suppressive capacity of intestinal Treg cells. On the contrary, defects in AHR result in aberrant Treg cell function and increased secretion of pro-inflammatory cytokines IFN-γ and IL-17A, thereby exacerbating colitis (59). Tr1 cells, a subset of Treg cells that do not rely on the transcription factor *Foxp3* but produce high levels of IL-10, are induced to differentiate by IL-27. This process involves the *STAT3* signaling pathway, which upregulates AHR expression. AHR, in turn, cooperates with the transcription factor *c-Maf* to promote the expression of Tr1 molecules such as IL-10, IL-21, and CD39 (60). The intestinal inflammatory microenvironment of IBD leads to reduced numbers and functional defects of Tr1 cells, and the AHR signaling pathway may be disrupted, resulting in insufficient secretion of anti-inflammatory factors like IL-10, weakening its ability to suppress pathogenic immune responses and ultimately leading to uncontrolled inflammation (61). Notably, AHR also promotes the conversion of Th17 cells into Tr1 cells, a mechanism that may help alleviate immunopathologic injury and facilitate the resolution of inflammation (62).

Intestinal epithelial lymphocytes (IELs) are special T cells that reside between the IECs. Despite their diversity in developmental origin and phenotypic characteristics, IELs uniformly exhibit core features including tissue residency, a combination of adaptive and innate immune functionalities, cytotoxic effects, and restricted TCR diversity. Collectively, these attributes contribute to a finely regulated defense system at the epithelial barrier (63). It is precisely through the regulation of IELs’ development, proliferation, and function that AHR maintains this intestinal immune system. Studies have shown that AHR deficiency results in a marked decrease in IEL populations and heightened vulnerability of the intestinal epithelium to damage (28). In addition, AHR activation can induce the differentiation of IELs into T cells with immunomodulatory properties, a process that is enhanced by indole derivatives generated through the metabolism of Trp by *Lactobacillus reuteri*, which act as AHR ligands (64). In a DSS-induced colitis model, administration of the AHR agonist FICZ was shown to inhibit apoptosis of CD8ααTCRαβ IELs by upregulating the expression of IL-15 receptor on the surface of IELs, thereby mitigating intestinal inflammation (65).

Macrophages contribute to intestinal inflammation in IBD through a dysregulated balance between M1and M2 phenotype polarization (66). The classical pro-inflammatory M1 phenotype is induced by stimuli such as IFN-γ and LPS, which potentiate Th1 immune responses and microbial clearance via secretion of IL-6, TNF-α, and reactive oxygen species (ROS). However, excessive M1 activation can damage the integrity of the intestinal epithelial barrier. Conversely, the anti-inflammatory M2 phenotype, stimulated by IL-4 and IL-13, releases IL-10 and enhances arginase activity, thereby suppressing inflammation and promoting tissue repair (67). Although the relationship between AHR and macrophages has not been fully elucidated, it is generally accepted that AHR deficiency disrupts polarization homeostasis, leading to abnormally elevated M1 marker expression and suppression of M2-related gene expression (68). Proposed mechanisms include (1): regulation of metabolic reprogramming by inhibiting M1-related glycolysis and promoting M2-associated oxidative phosphorylation (69) (2); activation of the Nrf2 antioxidant pathway to decrease ROS production and limit over activation of M1 (70) (3); enhancement of M2 marker expression, including *Arg1*, *Ym1*, and other markers, via increased signal transducer and activator of *STAT6* phosphorylation in synergy with IL-4/IL-13 signaling, alongside upregulation of *KLF4* and *PPARγ* transcription factors (71). Recent investigations have found that FICZ alleviates intestinal inflammation by promoting M2 macrophage polarization, with its protective effect potentially linked to upregulation of *IRF4* expression, thereby offering new insights into CD’s pathogenesis (72). In conclusion, ligand-activated AHR can induce macrophage conversion to the M2 phenotype, promote anti-inflammatory responses, and alleviate intestinal inflammation.

Dendritic cells (DCs), which are integral to antigen presentation and the differentiation of immune cells, represent a key target population for AHR ligands. Studies indicate that AHR deficiency results in metabolic disturbances and impaired DCs function, leading to an imbalance between Treg cells and Th17 cells, thereby exacerbating colitis. AHR contributes to the therapeutic modulation of IBD by regulating the immunomodulatory activities of DCs. On the one hand, AHR activation induces DCs to express *IDO1*/*IDO2*, which catalyze the conversion of Trp to kynurenine. This metabolic pathway promotes Treg cells differentiation and inhibits Th1/Th17-mediated inflammation via the AHR/*STAT3* signal axis (73). Meanwhile, AHR signaling also suppresses DCs' secretion of IFN-γ and upregulates IL-10 production, thereby reversing intestinal immune imbalance. On the other hand, tolerogenic dendritic cells (TolDCs), characterized as semi-mature cells with low expression of co-stimulatory molecules CD80 and CD86, are capable of triggering the proliferation and differentiation of Treg cells. Activation of AHR by FICZ has been shown to alleviate murine colitis through the induction of TolDCs. Further studies demonstrated that AHR-activated TolDCs increased Treg cell populations in DC-T cell co-culture models, suggesting AHR-mediated regulation of TolDCs attenuates DSS-induced colitis by reestablishing the Th17/Treg equilibrium (74). Additionally, AHR signaling mediates the production of anti-inflammatory cytokines by promoting the accumulation of Helios^+^ Treg cells and activating MHC II-expressing epithelial cells, thereby suppressing intestinal inflammation (75).

Beyond the aforementioned immune cells, the anti-inflammatory regulatory effects of the AHR signaling pathway in neutrophils, B cells, and natural killer cells (NK cells) have also been confirmed (76, 77). However, the specific molecular mechanisms underlying these effects in IBD remain to be fully elucidated. Recent studies have found that quercetin, a natural flavonoid compound and AHR agonist, can upregulate the expression of the AHR-ARNT complex in neutrophils, effectively inhibiting the formation of neutrophil extracellular traps and subsequently improving the inflammatory microenvironment within the colon (78). In summary, the multidimensional and multilayered regulation exerted by AHR on the functional state and dynamic balance of various immune cell subpopulations provides a critical molecular foundation for targeted therapeutic interventions aiming at restoring immune homeostasis in IBD.

#### 4.2.2 Mediating cytokine network remodeling

IL-22 is a pivotal cytokine involved in preserving the integrity of the intestinal barrier and promoting epithelial cell repair, thereby playing a significant role in the pathogenesis of IBD. Preclinical investigations utilizing DSS-induced colitis models have demonstrated that a deficiency in IL-22 or its receptor exacerbates intestinal inflammation. Conversely, administration of exogenous IL-22, such as IL-22-Fc fusion protein, has been shown to decrease intestinal permeability and promote mucosal healing (79). Notably, although elevated IL-22 levels are observed in certain IBD patients, the cytokine’s potential protective effects are often offset by the concurrent upregulation of its natural antagonist, IL-22-binding protein (80). The intestinal production of IL-22 is mediated by various immune cells, including ILC3, γδ T cells, Th17 cells, and CD4^+^ T cells (81), and the integrity of the AHR signaling pathway is critical for this process. AHR can directly bind to the DRE element within the IL-22 promoter region and synergistically interact with retinoic acid-related orphan receptor gamma t (*RORγt*) and *STAT3* to enhance IL-22 transcription. This molecular interplay contributes to the restoration of intestinal epithelial barrier repair and attenuation of intestinal inflammation (47, 82). Experimental evidence indicates that AHR-deficient mice exhibit markedly diminished IL-22 expression (83), impaired development of *RORγt*^+^ ILC3 cells, and a concomitant expansion of intestinal Th17 cells, underscoring the key role of AHR in balancing ILC3 and Th17 cell responses. The therapeutic potential of AHR agonists has also been validated in multiple experimental colitis models; for instance, treatment with FICZ activates AHR, leading to partial upregulation of IL-22 and downregulation of pro-inflammatory factors such as IFN-γ, IL-17α, and TNF-α, thereby ameliorating colitis induced by TNBS, DSS, and T cell transfer (26). Clinical data further suggest that FICZ treatment reduces IFN-γ levels and promotes IL-22 production in monocytes within the intestinal lamina propria of IBD patients. Although it is widely believed that IL-22 primarily exerts protective effects in inflammatory bowel disease, its pro-inflammatory properties and pro-proliferative effects in chronic inflammation have been confirmed in studies (84). Moreover, given the variations in AHR dosage, ligand types, and activation pathways, modulating the AHR-IL-22 signaling pathway may also trigger inflammatory responses (85). Therefore, the regulatory mechanisms of the AHR-IL-22 pathway require further in-depth investigation, and precise modulation of this signaling axis holds promise as a novel therapeutic strategy for promoting mucosal repair in IBD.

IL-10, a key anti-inflammatory cytokine regulating the AHR signaling pathway, is modulated through a dual mechanism mediated by AHR within specific immune cell populations. This regulation encompasses both direct transcriptional control of the IL-10 gene and the modulation of differentiation processes in IL-10-secreting immune cells. A variety of immune cell types, including monocytes, macrophages, dendritic cells, NK cells, and subsets of T lymphocytes, produce IL-10 via an AHR-dependent pathway (86). Fluctuations in IL-10 levels may arise from intrinsic regulatory mechanisms governing cytokine synthesis or from alterations in the population size of IL-10-producing cells. Importantly, AHR signaling exerts systematic control over IL-10 expression by influencing the differentiation of ILCs, Th17, Treg, and Tr1 (53). Among these, activation of AHR significantly promotes the differentiation of Tr1 and *Foxp3^+^* Treg cells, concomitantly upregulating their IL-10 production (87). Gene knockout models have demonstrated that AHR deficiency compromises IL-10 secretion by NK cells (88), and similarly, IL-10 expression is significantly diminished in AHR-/- macrophages following LPS stimulation (89). Pharmacological studies have further shown that AHR agonists, such as indigo extract and ITE, alleviate experimental colitis by inducing IL-10 and IL-22 production in colonic ILCs or by promoting the generation of IL-10^+^ Treg cells, respectively (90, 91). At the molecular level, AHR cooperates with *c-Maf* to directly regulate IL-10 gene expression in Tr1 cells (92). At the same time, Trp metabolites, including Kyn and indole-3-propionic acid (IPA), enhance the expression of the IL-10 receptor alpha subunit in IECs through AHR activation (27, 93).

In addition to modulating the above anti-inflammatory factors, the AHR mitigates intestinal inflammation by suppressing the overexpression of pro-inflammatory factors such as TNF-α, IL-1β, and IL-6 (31). Studies have shown that AHR can directly interact with the *NF-κB p65* subunit, thereby blocking its translocation into the nucleus and then downregulating the expression of factors such as TNF-α and IL-6 (94). Experimental studies in animal models have confirmed that the administration of the AHR agonist TCDD significantly reduces IL-23 and IL-1β levels in the colonic tissue of mice with DSS-induced colitis (95). Conversely, AHR gene knockout mice exhibit sustained release of inflammatory cytokines. Finally, the immunomodulatory function of AHR is closely associated with the gut microbiota (96); metabolites derived from the microbiota act as endogenous ligands for AHR, activating it to modulate host immune responses. However, dysbiosis commonly observed in patients with IBD results in a deficiency of these endogenous AHR ligands, thereby exacerbating disruptions in immune homeostasis (97, 98). This microbiota-immune axis interaction highlights the pivotal role of AHR as a central hub connecting gut microbiota ecology with the maintenance of host immune homeostasis.

### 4.3 Mediating microbe-host interactions

Analysis of the gut microbiota of IBD patients showed significant alterations in microbial composition compared to healthy populations, characterized by a reduced ratio of *Firmicutes/Bacteroidetes*, decreased microbial diversity, and an imbalance between anti-inflammatory and pro-inflammatory microbiota, confirming the central role of microbial dysbiosis in the pathophysiology of IBD (99). AHR maintains intestinal microbial homeostasis through multiple mechanisms, and its functional defects are closely related to IBD.

#### 4.3.1 AHR shapes the gut microbiota

Defects in the AHR signaling pathway have been implicated in mediating microbiotal dysbiosis and perpetuating a vicious cycle of chronic inflammation. The expression of AHR is essential for the colonization and maintenance of the gut microbiota, with its activity influencing the proportion of specific bacterial populations within the intestine, thereby remodeling the microbial community structure (100). Dietary intake rich in AHR ligands can significantly modify the gut microbiota composition in mice, conferring protective effects on intestinal health (101). However, exposure to dietary TCDD, through activation of the AHR signaling pathway, modulates the *Firmicutes* to *Bacteroidetes* ratio, exacerbating inflammation (102). In AHR-deficient mice subjected to diets devoid of AHR ligands, a marked reduction in the abundance of *Bacteroidetes*, *Actinobacteria*, and *Tenericutes* was observed relative to wild-type counterparts; however, supplementation with ligand-rich diets resulted in an upregulation of *Firmicutes* and a concomitant downregulation of *Bacteroidetes* abundance (103). Furthermore, neuron-specific defects in AHR have been associated with delayed colonic transit, retention of intestinal contents, proliferation of pathogenic bacteria, inhibition of beneficial bacterial colonization, and destabilization of microbiota composition (11). AHR contributes to intestinal homeostasis by regulating bacterial load, promoting the secretion of antimicrobial peptides, and preventing bacterial translocation. Notably, diminished AHR activity correlates with reduced antimicrobial peptide secretion (104); administration of the AHR agonist FICZ effectively restores this secretion via activation of the AHR/IL-22/*STAT3* signaling axis. Meanwhile, AHR also inhibits the Notch1 signaling pathway through non-enzymatic interaction with *IDO1* in IECs, facilitating differentiation of secretory cells and enhancing mucus layer thickness. This enriches the mucus-associated beneficial microbiota, such as *Akkermansia muciniphila*, while inhibiting colonization by pathogenic bacteria to achieve the regulation of the composition and function of the intestinal microbiota (105). Moreover, AHR is capable of recognizing quorum-sensing molecules secreted by pathogenic bacteria, including Pseudomonas aeruginosa, enabling surveillance of infections; aberrations in AHR function compromise the host's ability to adapt to disturbances in gut microbial communities (106).

#### 4.3.2 Feedback regulation by microbial-derived AHR ligands

Research has found that levels of microbial-derived AHR ligands and AHR activity are reduced in fecal samples obtained from IBD patients (107). Numerous investigations have emphasized the key role of the gut microbiota in supplying AHR ligands (108). A variety of natural ligands generated through gut microbial metabolism serve as critical signaling molecules for AHR activation, forming the core of the “microbe-ligand-AHR” regulatory axis. Trp, an essential amino acid, is integral to this axis; it undergoes metabolism via host enzyme systems and specific gut microbiota to yield various AHR ligands. Among them, microbial-derived Trp metabolites, predominantly indoles and their derivatives, are recognized as reliable sources of AHR ligands (109). The gut microbiota directly regulates both the composition and concentration of these metabolites, which in turn influence epithelial barrier function, maintain the balance between pro-inflammatory and anti-inflammatory cytokines, and resist pathogen colonization through AHR activation (110, 111). Studies have shown that IEt, IPyA, and I3A, which are generated by microbial metabolism of Trp, can activate AHR and suppress phosphorylation of myosin IIA and ezrin. This mechanism preserves the integrity of the apical junctional complex within the intestinal epithelium and significantly improves DSS-induced colitis in mice (96). Activation of the Trp-AHR pathway induces the expression of downstream cytokines, including IL-22 and IL-17, thereby contributing to the regulation of microbiota-host homeostasis. Specifically, I3C increases IL-22 production, mitigates colitis-related microbiota dysbiosis (112), promotes Treg cells differentiation, inhibits Th17 cell differentiation, and reduces IL-17 production to alleviate colitis (113). Additionally, IAld, a Trp metabolite synthesized by Lactobacillus species and an AHR ligand, activates ILC3s, thereby strengthening mucosal defense against opportunistic pathogens such as *Candida albicans* (29).

SCFAs are the primary products of gut microbiota fermentation of dietary fiber, with acetate, propionate, and butyrate being the most abundant in the gastrointestinal tract, offering multiple health benefits to the host (114). Among them, butyrate has attracted considerable attention owing to its strong anti-inflammatory and antioxidant properties (115). Previous studies have proposed that butyrate functions as a ligand for AHR, thereby activating the AHR pathway and regulating the expression of its downstream target genes, such as *CYP1A1* and *AHRR*, indicating its potential involvement in diseases like IBD. Additionally, butyrate can upregulate AHR and HIF1α expression by activating GPR41 and inhibiting HDAC activity, thereby promoting the production of IL-22 by CD4^+^ T cells and ILCs and protecting the intestine from inflammatory damage (116). However, whether butyrate directly binds to AHR remains controversial. Some studies suggest that butyrate is not a direct ligand for AHR but rather acts by inhibiting HDAC activity, remodeling chromatin structure, and enhancing the binding capacity of AHR to target gene promoters, thereby synergistically activating the AHR pathway with microbial-derived Trp metabolites (117). Additionally, phenolic compounds produced by gut microbiota metabolism of dietary polyphenols may directly bind to or inhibit HDAC activity, thereby regulating AHR activity through mechanisms such as modulating the AHR/IL-22 axis, enhancing intestinal barrier function, and inhibiting Th17 cell differentiation, thereby alleviating intestinal inflammation (118). However, the precise mechanisms underlying these effects warrant further investigation.

#### 4.3.3 The AHR-microbiota axis in IBD

As can be seen, AHR achieves bidirectional regulation via the “microbiome-ligand-AHR-host” feedback loop. Specifically, AHR actively shapes the intestinal microenvironment while concurrently integrating microbial ligand signals to coordinate the host's immune response. This feedback mechanism underpins the maintenance of intestinal homeostasis, and its dysfunction leads to diminished host resilience to microbiota perturbations, imbalance of immune homeostasis, and barrier disruption, thereby perpetuating a vicious cycle contributing to the development of IBD (119). Given the central role of this axis in IBD pathogenesis and its therapeutic potential, current intervention strategies focus on the remodeling of the beneficial microbiota composition alongside targeted modulation of AHR signaling pathways.

Therapeutic approaches targeting the microbiota have demonstrated potential in alleviating IBD through the regulation of AHR activity. Recent investigations have identified several probiotics capable of engaging AHR to regulate intestinal function and attenuate intestinal inflammation. *Akkermansia muciniphila* has emerged as a promising probiotic that prevents colitis by activating the AHR-Trp signaling pathway (120). Additionally, strains of *Bifidobacterium bifidum*, especially FL-276.1 and FL-228.1, have been shown to promote AHR-mediated pathways, thereby improving DSS-induced colitis and protecting barrier function (108). *Lactiplantibacillus plantarum*, a probiotic commonly found in fermented foods, efficiently metabolizes Trp to produce indole-3-lactic acid (ILA) (121), which activates the AHR*CYP1A1*/IL-22 signaling cascade. This activation significantly alleviates colon inflammation in mice and repairs the intestinal barrier. Furthermore, animal studies indicate that *Bacteroides thetaiotaomicron* can activate AHR and modulate the differentiation profile of CD4^+^ T cells in a mouse DSS-induced colitis model system, suggesting its potential therapeutic relevance for IBD treatment. Similar results have been reported in *Lactobacillus bulgaricus* OLL1181 (122). FMT has emerged as a novel therapy for IBD, with mechanisms that are not yet fully understood but are closely related to AHR pathway activation. Clinical trials show that 61.29% of patients with active UC achieved clinical remission after FMT (123). Complementary animal studies confirm that FMT significantly increases the abundance of *Bifidobacterium* and *Lactobacillus* species, concomitantly elevating AHR expression and anti-inflammatory cytokine levels within colonic tissues, thereby restoring intestinal homeostasis and alleviating colitis (30). Importantly, modified FMT, pretreated with *Lactobacillus plantarum* GR-4, enhances the production of ILA and IAA via Trp metabolism, upregulates IL-22 and tight junction protein synthesis through AHR signaling pathways, and markedly improves colitis remission rates compared to traditional FMT, highlighting the effectiveness of FMT in IBD via the AHR axis and the optimizing effect of probiotic pretreatment (124).

**Figure 3:**
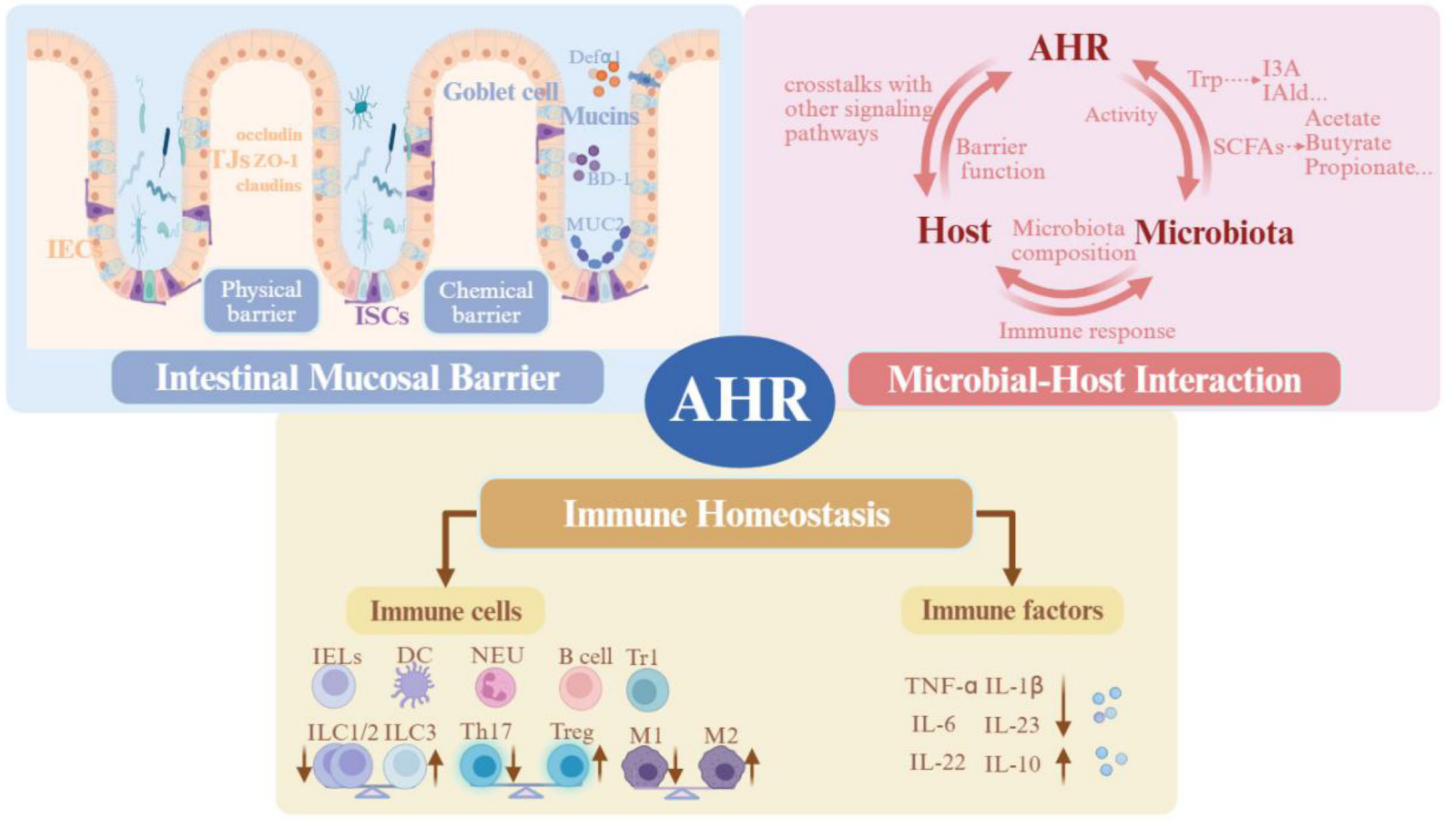
Key mechanisms of AHR regulation in IBD. AHR modulates the progression of IBD through three coordinated mechanisms: intestinal mucosal barrier repair, microbiota-host interactions, and immune homeostasis. AHR Activation regulates the functions of IECs and ISCs, promotes the expression of TJs (ZO-1, claudins, Occludin) to restore the epithelial physical barrier, induces goblet cell proliferation and MUC2 secretion to enhance the chemical barrier, and upregulates AMPs to reinforce the biological barrier. Gut microbiota-derived metabolites serve as endogenous AHR ligands, forming a positive feedback loop that regulates microbial composition and metabolic homeostasis. Immunologically, AHR activation modulates the functionality of various immune cells, including macrophage polarization, Th17/Treg balance, IELs, and DCs, while simultaneously suppressing pro-inflammatory cytokines and promoting the expression of IL-22 and IL-10. By integrating these three core aspects-intestinal barrier, microbiota, and immunity-AHR represents a promising therapeutic target for IBD.

In addition to microbiological interventions, dietary strategies-such as the Mediterranean diet or supplementation with exogenous AHR ligands may ameliorate the pathological progression of IBD by modulating the composition of intestinal microbiota and activating the AHR pathway (118, 125). Although the therapeutic potential of AHR is gradually acknowledged, several challenges impede its clinical application as a treatment for IBD. While synthetic AHR agonists have shown considerable efficacy in animal models (126), concerns regarding their immunotoxicity and carcinogenicity severely limit their use in clinical settings (9, 127). Therefore, there is a pressing need to develop effective intervention strategies that leverage natural AHR ligands and indirect regulation of the microbiota, thereby optimizing therapeutic benefits while ensuring clinical safety. (**Figure 3**).

## 5 Therapeutic potential of natural products targeting AHR

Natural products have emerged as a crucial resource in the development of therapeutics for IBD, owing to their diverse chemical structures, multi-target regulatory properties, and significant therapeutic potential. The synergistic advancement in contemporary chemical analysis and pharmacological technologies has propelled research on natural products toward greater standardization and systematization, providing new perspectives for drug discovery. Considering the central role of AHR in maintaining intestinal homeostasis and its participation in the pathogenesis of IBD, it has been identified as a highly promising therapeutic target. To date, studies indicate that various natural products can exert anti-inflammatory, barrier repair, and microbiota homeostasis reestablishment effects through activation of the AHR signaling pathway. It should be noted that the “natural products” focused on in this paper mainly include three types of research objects: pure compounds isolated and purified from natural resources, natural extracts that remain in a multi-component complex state, and bioactive molecules metabolized by gut microbiota. Although they differ in existing forms and chemical complexity, they all originate from nature and have demonstrated significant scientific value and application potential in the research on AHR-mediated IBD regulation mechanisms and therapeutic exploration. This review summarizes the mechanism of action and therapeutic potential of natural compounds, including phenolic compounds, alkaloids, terpenoids, polysaccharides, microbial metabolites, and other natural products that modulate IBD via AHR-related pathways. (**Table 2**).

**Table 2:** Natural products for treating IBD via AHR signaling pathways.

Classification	Product	Source	Model	Administration	Concentration/vehicle	Mechanism	Ref.
Phenolic							
Flavonoids	Baicalein	*Scutellaria baicalensis*	DSS-induced mice, T cell differentiation model; DSS-induced mice, LPS-stimulated Caco-2 cells	Orally (Gavage) Gavage	10, 20, 40 mg/kg - 10, 20, 40 mg/kg deionized water	Activated AHR to regulate the balance of Th17/Treg cells; AHR-IL-22 pathway	(128, 129)
	Wogonin	*Scutellaria baicalensis*	DSS-induced mice, MNK3 cells, NCM460 cells	Gavage	20 mg/kg -	Activated AHR to regulate the plasticity of ILC3/ILC1	(130)
	Quercetin	*Quercus*	DSS-induced mice, PMA-stimulated THP-1 cells and Caco-2 cells	Gavage	20, 50 mg/kg deionized water	Activated AHR to upregulate TJs (e.g., ZO-1, Claudin-1)	(131)
	Cardamonin	*Alpinia katsumadai*	DSS/TNBS-induced colitis in mice, cellular assay *in vitro*	Gavage Per rectum	15, 30, 60 mg/kg; - 60mg/kg	Activated AHR-Nrf2/NQO1 pathway to inhibit NLRP3 inflammasome activation	(132)
	Isomonyxin	Legumes, grains, and fruits	DSS-induced mice, TNF-α-stimulated IEC cells	Oral	25, 50, 100 mg/kg DMSO + PBS mixture	Activated AHR to inhibit NF-κB activation	(133)
	Myricetin	*Myrica*	DSS-induced mice, molecular docking/cellular validation	Gavage	40, 80 mg/kg -	Activated AHR to regulate the balance of Th17/Treg cells	(134)
	Alpinetin	*Alpinia katsumadai* and *Alpinia officinarum*	DSS-induced mice, T cell differentiation model *in vitro*; DSS-induced mice, an IECs model *in vitro*	Gavage Gavage	7.5, 15, 30 mg/kg 0.5% CMC-Na 30 mg/kg 0.5% CMC-Na	Activated AHR to recover Th17/Treg balance Activated AHR to enhance intestinal barrier function	(135, 136)
	Pelargonidin	Ripe raspberries and strawberries	DSS-induced mice, TNBS-induced mice	Gavage	5, 10mg/kg -	Activated AHR to regulate immune homeostasis	(137, 138)
Other phenols							
	Magnolol	*Magnolia officinalis*	DSS-induced mice	Gavage	5, 10, 15 mg/kg Saline	Rejuvenated Trp metabolism to trigger AHR activation and ameliorate inflammation	(139)
	Theabrownin	*Fuzhuan Brick Tea*	DSS-induced mice, a multi-omics analysis model; DSS-induced mice, a mechanism validation model	Gavage Gavage	400 mg/kg; Water 25, 50, 100 mg/kg Saline	Reshaped gut microbiota and Trp metabolism to activate AHR and repair the intestinal barrier; Modulated gut microbiota-I3A-AHR signaling pathway	(140, 141)
	Alpha-tocopherol quinone	Vitamin E	DSS/TNBS-induced mice, Caco-2/gene transfection cell model, T cell transfer model	Gavage	50 mg/kg -	Activated AHR to increase CLDN3; Activated AHR to suppress the *STAT3*-NF-κB signaling	(142, 143)
Alkaloids							
	Berberine	*Coptis chinensis*, *Phellodendron amurense*	DSS-induced mice, Caco-2 cells	Gavage	40mg/kg Water	Improved microbiota metabolites to activate AHR and improve intestinal barrier function	(2)
	Coptisine	*Coptis chinensis* *Phellodendron amurense*	DSS-induced mice, AHR knockout organoid, and mice	Gavage	25, 50 mg/kg -	Activated AHR to upregulate TJs and inhibit NF-κB/ROS activation	(144)
	Norisoboldine	*Cocculus orbiculatus*, *Lindera aggregata*	DSS-induced mice TNBS-induced mice, LPS/ATP-stimulated THP-1 cells	Gavage/Per rectum; Gavage	40mg/kg - 20, 40 mg/kg -	Activated AHR to promote Treg cells differentiation; Regulated AHR-Nrf2/ROS signaling pathway to inhibit NLRP3 inflammasome activation	(70, 145)
	Tetrandrine	*Stephania tetrandra*	DSS-induced mice, TNF-α-stimulated Caco-2 cells	Gavage	10, 20, 40 mg/kg HCl + NaOH	Activated AHR to elevate the expression of TJs	(146)
Terpenoids							
	Ganoderic Acid A	*Ganoderma lucidum*	DSS-induced mice, FMT	Gavage	20 mg/kg Water	Activated AHR to induce IL-22 production	(147)
	Ginsenoside CK	*Panax ginseng*	DSS-induced mice,	Gavage	80mg/kg saline	Modulated the microbiota-Trp metabolite-AHR axis	(148)
Polysaccharides							
	β-Glucan	*Saccharomyces cerevisiae*	DSS-induced mice	Gavage	400mg/kg PBS	Regulated *L. johnsonii*-ILA-AHR axis	(149)
	Kiwifruit polysaccharide	*Actinidia* spp.	DSS-induced mice, antibiotic clearance, microbiota model	Gavage	200, 400 mg/kg Saline	Regulated Trp metabolism to activate AHR and enhance IL-22 production and TJs expression	(150)
	Astragalus polysaccharide	*Astragalus membranaceus*	DSS-induced mice, *Becn1* knockout mice, LPS-induced Caco-2 cells	Gavage	100, 200, 400 mg/kg PBS	Activated AHR to upregulate autophagy, alleviating inflammation	(151)
	Fucoidan	Phaeophyceae	Acetic acid-induced mice,	Gavage	150mg/kg -	Upregulated AHR to activate antioxidant and anti-inflammatory systems	(152)
	Turmeric polysaccharide	*Curcuma longa*	DSS-induced mice	Dietary supplementation	8% w/w -	Regulated microbiota and metabolites to activate AHR and IL-22 production and TJs	(153)
Other							
Sugarderivatives							
	L-Fucose	Phaeophyceae	DSS-induced mice	Gavage	250mm/kg water -	Regulated AHR-IL-22 pathway in LPMCs	(154)
	Fructooligosaccharides	*Cichorium intybus*, *Asparagus officinalis*	DSS-induced mice	Gavage	400mg/kg -	Regulated IAA/IPA-AHR-IL-22 axis	(155)
Polyamine							
	Spermidine	Soya, metabolically produced by microbiota	DSS/TNBS-induced mice; TNF-α/IFN-γ-stimulated Caco2 cells	Drinking	5, 20 mM; water	Regulated AHR-Nrf2/*STAT3* signaling pathway	(156)
Natural extract							
	Indigo Naturalis	*Strobilanthes cusia*, *Polygonum tinctorium*, *Isatis tinctoria*	DSS/TNBS-induced mice, cell culture *in vitro*	Gavage	600, 120`300, 60 mg/kg; Water	Activated AHR to upregulate IL-10 and IL-22; Activated AHR to induce ILC3-IL-22 production	(91, 157)
	*Schisandra chinensis*	*Schisandra chinensis*	DSS-induced mice	Gavage	1.95, 3.9 g/kg; -	Regulated Trp metabolism to activate AHR and inhibit NF-κB and enhance TJs	(158)

### 5.1 Phenolic compounds

Phenolic compounds are widely present in various sources of the daily diet, including vegetables, fruits, beverages such as coffee, tea, and wine, as well as many medicinal plants, making them one of the most abundant phytochemicals in the human diet (159). Literature reviews indicate that phenolic compounds possess multiple bioactivities, including cardiovascular protection, antioxidant, neuroprotective, anticancer, and anti-inflammatory effects (118). These activities also partly explain their therapeutic value in traditional Chinese medicine (160). Studies have shown that phenolic compounds can activate the AHR signaling pathway either by directly binding to AHR as ligands or by increasing endogenous AHR ligands—such as Trp metabolites—through modulation of the gut microbiota. AHR activation upregulates TJs to reinforce the intestinal barrier, promotes the release of protective cytokines like IL-22, and restores immune balance (e.g., Th17/Treg) while suppressing inflammatory pathways including NF-κB and NLRP3, collectively alleviating intestinal inflammation and promoting tissue homeostasis.

#### 5.1.1 Flavonoids

Baicalein, the principal flavonoid constituent of the traditional Chinese medicine *Scutellaria baicalensis*, exhibits multiple bioactive effects, including anti-inflammatory, antioxidant, antitumor, and intestinal epithelial barrier-repairing functions (161, 162). Previous investigations have revealed that baicalein alleviates intestinal inflammation by modulating the balance between Th17 and Treg cells via activation of the AHR signaling pathway, suggesting its therapeutic potential for UC (128). Subsequent research has further demonstrated that baicalein reduces intestinal inflammation, restores the expression of colonic TJs such as ZO-1 and Occludin, and decreases intestinal permeability. The underlying protective mechanism is posited to involve AHR activation, its nuclear translocation, and consequent enhancement of IL-22 production by ILC3, which collectively maintain tight junction integrity and improve the intestinal barrier (129).

Analogously, wogonin, another flavonoid isolated from *Scutellaria baicalensis*, has been characterized as an AHR ligand with demonstrated efficacy in ameliorating acute colitis and suppressing colorectal carcinogenesis (163, 164). Studies demonstrate that wogonin alleviates DSS-induced chronic colitis. As an exogenous ligand, wogonin directly interacts with the AHR ligand-binding domain, facilitating its nuclear translocation within ILC3s to regulate the plasticity between ILC3 and ILC1 subsets. This modulation results in enhanced IL-22 secretion concomitant with reduced IFN-γ production, promoting epithelial barrier repair. Furthermore, wogonin influences the gut microbiota composition, leading to an increased generation of microbially derived metabolites that serve as endogenous AHR ligands. These metabolites collectively activate AHR signaling pathways, fine-tuning the plasticity of ILC3/ILC1 (130).

Quercetin, a widespread flavonoid extensively distributed within the plant kingdom, is mainly present in the bark and leaves of the *Quercus* species, as well as in certain fruits and vegetables. It exhibits notable anti-inflammatory, antioxidant, and antimicrobial properties (165, 166). A meta-analytical review assessing the therapeutic potential of quercetin in IBD indicates that its protective effects against IBD pathogenesis may stem from multimodal actions, including anti-inflammatory activity, attenuation of oxidative stress, cytoprotection, reinforcement of the barrier, and modulation of the microbiota (167). *In vivo* studies involving oral administration of quercetin to mice with DSS-induced colitis showed that quercetin alleviated colitis by restoring tight junction integrity via an AHR-dependent mechanism. *In vitro* experiments using Caco-2 cells revealed a dose-dependent upregulation of TJs ZO-1 and Claudin-1 following quercetin treatment. Concurrently, quercetin activated AHR, evidenced by enhanced expression of *CYP1A1* and promoted nuclear translocation of AHR. Critically, the application of the AHR antagonist CH223191 reversed the therapeutic effects of quercetin, inhibiting both AHR activation and the enhancement of TJ proteins. These findings confirm that quercetin repairs intestinal epithelial barrier damage through AHR activation (131).

Cardamonin, a flavonoid derived from plants of the *Zingiberaceae* family, such as *Alpinia katsumadai*, has been documented to possess significant anti-inflammatory and immunomodulatory properties (168, 169). Experimental studies demonstrate that cardamonin markedly ameliorates colitis in murine models induced by both DSS and TNBS. Mechanistic investigations further validate that its therapeutic effects are mediated via activation of the AHR/Nrf2/NQO1 signaling axis, which subsequently suppresses the activation of the NLRP3 inflammasome (132).

Isovitexin (ISO), a natural flavonoid glycoside widely found in numerous plants, possesses demonstrable anti-inflammatory effects (170, 171). Although investigations on the role of ISO in colitis are limited, existing studies have indicated that ISO treatment alleviates symptoms in mice with DSS-induced colitis, demonstrating its protective potential against this disorder. Mechanistic analyses have shown that ISO dose-dependently increases AHR expression and inhibits NF-κB activation. These findings suggest that ISO's protective efficacy is primarily achieved through AHR activation, which subsequently suppresses inflammation and protects intestinal barrier integrity (133).

Myricetin, a flavonoid characterized by three adjacent hydroxyl groups, has been shown to possess antioxidant, anticancer, and anti-inflammatory properties (172, 173). Previous studies established its ability to mitigate murine colitis by regulating Th17/Treg balance, but the underlying mechanism remains unclear. Recent studies have reported that Myricetin counteracts DSS-induced abnormalities in amino acid metabolism, specifically within the Trp, phenylalanine, and tyrosine pathways. Mechanistically, myricetin interacts with the ligand-binding domain of AHR, thereby activating AHR and its catalytic site, which leads to upregulation of cytochrome P450 genes *CYP1A1* and *CYP1B1*. This cascade restores Th17/Treg homeostasis and ultimately confers protection against DSS-induced colitis via activation of the AHR signaling pathway (134).

Alpinetin is a major bioactive flavonoid mainly derived from *Zingiberaceae* plants of the genus Alpinia and exhibits important therapeutic potential, including antimicrobial and antitumor properties (174). Studies have found that alpinetin alleviates inflammatory responses by inhibiting the TLR4 and NLRP3 signaling pathways in DSS-induced acute colitis, suggesting its promise as a therapeutic agent for colitis (175). Furthermore, alpinetin significantly improves murine colitis by restoring Th17/Treg cell balance—an effect mechanistically related to AHR activation and regulation of miR-302/DNMT-1/CREB signaling axis (135). Additionally, alpinetin activates AHR to induce E3 ubiquitin ligase-mediated proteasomal degradation of the histone methyltransferase SUV39H1, thereby downregulating SUV39H1 expression. This process promotes colonic epithelial cell autophagy via regulation of the tuberous sclerosis complex 2 (*TSC2*)-mTORC1 signaling axis, ultimately improving UC (136). Notably, AHR antagonists significantly abolish alpinetin's protective effects on the intestinal mucosal barrier, confirming the AHR-dependent nature of its therapeutic actions.

Pelargonidin is a water-soluble anthocyanin that forms the red or orange color of berries, including ripe raspberries and strawberries, as well as blueberries, blackberries, and cranberries, and others (176). In nature, it exists in the form of glycosylated derivatives. Ghattamaneni et al. reported that dietary pelargonidin-3-glucoside alleviates symptoms and reduces intestinal inflammation in rats with DSS-induced chronic IBD (177). They proposed that the underlying mechanism may involve activation of the AHR pathway and modulation of inflammatory signaling. This finding aligns with research by Biagioli and colleagues, who demonstrated that pelargonidin compounds ameliorate experimental colitis through AHR, dependent on direct ligand-receptor interaction (137). Furthermore, Zdeněk, by integrating existing studies, clearly proposed that pelargonidin compounds alleviate IBD via the AHR pathway, emphasizing the critical role of gut microbiota metabolism in determining local ligand concentration (138).

#### 5.1.2 Other phenolic compounds

Magnolol, primarily isolated from the dried bark of *Magnolia officinalis* and related species, exhibits diverse pharmacological activities, including anticancer, anti-stress, anti-anxiety, antidepressant, antioxidant, and anti-inflammatory properties (178). While magnolol's anti-inflammatory efficacy has been demonstrated in animal models of various inflammatory diseases, its specific impact on intestinal inflammation remains unvalidated (179, 180). Zhang et al. were the first to explore magnolol's effect on DSS-induced colitis, confirming its protective role against murine colitis (139). The underlying mechanism may involve enhanced AHR activation by increasing the production of Trp metabolites, which are suppressed during colonic inflammation. Furthermore, in the DSS-induced colitis model, magnolol ameliorated colitis symptoms, attenuated pro-inflammatory cytokine levels, and histopathological manifestations by modulating MAPK and NF-κB signaling pathways (181).

*Fuzhuan Brick Tea, a typ*e of dark tea produced through a specific microbial fermentation process from the leaves of *Camellia sinensis* (family Theaceae), is rich in polyphenols and exhibits notable anti-inflammatory and antioxidant properties (182). Theabrownin, a water-soluble pigment generated via the oxidation and polymerization of tea polyphenols during microbial fermentation, constitutes a key component of *Fuzhuan Brick Tea* (183). Studies have shown that Theabrownin can alleviate colonic inflammation by inducing the proliferation of Trp metabolism-associated gut bacteria, especially Lactobacillus spp. Subsequent microbial metabolism increases the production of protective Trp metabolites, such as IAA and IAld. These metabolites activate the AHR pathway, thereby enhancing the production of IL-22 to repair the intestinal epithelial barrier (140). In a separate study, Fuzhuan Brick Tea Polysaccharide (FBTP) was found to exert a preventive effect against experimental colitis through the gut microbiota-AHR ligand production-immunity axis. This suggests that Theabrownin may serve as a potential next-generation prebiotic to improve intestinal epithelial homeostasis and ameliorate colitis through gut microbiota-dependent modulation of the AHR pathway (141).

Alpha-tocopherol quinone (α-TQ), a vitamin E-derived non-toxic and low-reactive quinone, maintains cell membrane integrity and regulates oxidative stress and cell death (184, 185). However, the molecular mechanisms underlying its diverse physiological functions are not completely elucidated. Research demonstrates that α-TQ upregulates CLDN3 expression through AHR-dependent pathways and inhibits CLDN2 through the Nrf2-SHP-*STAT3* signaling axis, significantly increasing trans-epithelial resistance and reducing permeability in Caco-2 cells, mice, and human colon tissue, effectively alleviating DSS/TNBS/T cell-induced colitis (142). Additionally, α-TQ activates AHR to inhibit the NF-κB/*STAT3* pathway and downregulate IL-6R in T cells, thereby blocking the IL-6-IL-17 inflammatory axis (143). Collectively, as a natural AHR activator, α-TQ synergistically enhances intestinal barrier function and regulates immune responses to improve inflammation, demonstrating its potential as a therapeutic agent for IBD.

### 5.2 Alkaloids

Alkaloids are a class of nitrogen-containing compounds primarily found in plants, mostly characterized by complex alkaline structures (186). They serve as important active components in traditional Chinese herbal medicines and exhibit various biological activities such as antibacterial, antitumor, and immunomodulatory effects. Literature indicates that many alkaloids can not only directly act as AHR agonists to activate this signaling pathway, but also promote the production of endogenous AHR ligands by modulating the gut microbiota, thereby exerting protective effects, including intestinal barrier repair, immune regulation, and anti-inflammatory actions.

Berberine is an isoquinoline alkaloid primarily derived from *Coptis chinensis* and *Phellodendron amurense*, possessing pharmacological activities including antibacterial, anti-inflammatory, analgesic, and antitumor effects. While studies indicate its therapeutic potential for IBD, its specific mechanism has not been fully defined (187). Research has shown that berberine ameliorates intestinal epithelial barrier dysfunction by upregulating ZO-1 and Occludin expression and mediating P-glycoprotein, contributing to its IBD efficacy (188, 189). Furthermore, in a DSS-induced rat colitis model, berberine significantly alleviated colonic inflammation. This protective effect was closely related to the restoration of microbial homeostasis: Berberine reversed DSS-induced depletion of Lactobacillus, Clostridium, and Bacteroides abundance. Consequently, enhanced microbial Trp metabolism modulated IAA, IPA, and IA levels, activating the AHR pathway to regulate TJs and protect barrier function (2).

In comparison, the homologous alkaloid coptisine also exhibits multiple biological activities, such as antibacterial and antitumor activity, as well as significant therapeutic potential for IBD (190, 191). Coptisine directly activates AHR by promoting the nuclear translocation and transcriptional regulation of cytochrome P450 enzyme *CYP1A1*, thereby enhancing AHR’s biological effects (144). In the TNF-α-stimulated HT-29 cell model, coptisine dose-dependently inhibited ROS generation and NF-κB activation, while upregulating tight junction protein expression, ultimately restoring intestinal epithelial barrier function and alleviating colonic inflammatory responses.

Norisoboldine (NOR), mainly extracted from the roots of *Cocculus orbiculatus* and *Lindera aggregata*, exhibits a remarkable ability to activate AHR, which is the core active ingredient for its anti-inflammatory effects (192). Previous experiments have shown that NOR can alleviate UC and induce the production of Treg cells, but the specific mechanism remains unclear (193). It was found that NOR could promote Treg differentiation through regulating the AHR-glycolysis axis and the subsequent NAD/SIRT1/SUV39H1/H3K9me3 signaling pathways, thereby exerting anti-UC effects (145). In addition, NOR can also improve TNBS-induced colitis in mice by regulating the AHR/Nrf2/ROS signaling pathway to inhibit NLRP3 inflammasome activation (70). Notably, NOR’s low oral bioavailability limits its potential for future development and application. To address this limitation, researchers have designed and synthesized a NOR derivative, which not only activates target gene expression through the AHR pathway but also enhances Treg cell differentiation (126). These findings provide valuable insights for designing novel AHR agonists targeting immune and inflammatory diseases.

Tetrandrine, a bisbenzylisoquinoline alkaloid primarily derived from the Chinese herb *Stephania tetrandra*, has been demonstrated to ameliorate DSS-induced colitis (194). Chu et al. investigated the impact of tetrandrine on intestinal mucosal barrier defects to elucidate its anti-colitis mechanism. They revealed that tetrandrine alleviates intestinal epithelial barrier impairment in colitis by upregulating the expression of the tight junction protein Occludin via the AHR–miR-429 pathway, suggesting its potential as a barrier-protective agent for colitis treatment (146).

### 5.3 Terpenoids

Terpenoids are one of the most important secondary metabolites in plants and have garnered widespread attention in recent years due to their significant and diverse biological activities, including anticancer, antioxidant, antiviral, and anti-inflammatory effects (195). Research indicates that the mechanism by which terpenoids regulate AHR primarily involves the “microbe-ligand-AHR” axis, which subsequently activates the AHR signaling pathway to exert therapeutic effects on IBD.

Ganoderic Acid A (GAA), a triterpenoid derived from the edible mushroom Ganoderma lucidum, has significant anti-inflammatory, antioxidant, anti-tumor, and hypolipidemic effects (196). Studies have shown that supplementing with GAA in DSS-induced colitis mice effectively prevents colitis, maintains the integrity of the epithelial and mucous layers, and regulates the gut microbiota. Furthermore, GAA significantly increased IAld levels through Trp metabolism, subsequently activating AHR and promoting IL-22 production, which helped maintain intestinal barrier function and improve IBD symptoms (147).

Ginsenoside compound K (CK), a triterpenoid sapogenin metabolite derived from *Panax ginseng*, is reported to possess therapeutic potential for metabolic syndrome, cardiovascular diseases, and exhibits antioxidant, anti-inflammatory, and antitumor activities (197, 198). Previous studies have demonstrated that CK is an effective anti-inflammatory natural product for treating IBD, but its mechanism of action remains unclear (199). Recent research further confirms that CK effectively alleviates DSS-induced IBD by modulating the gut microbiota and restoring intestinal barrier function. Moreover, CK activates AHR and upregulates *CYP1A1* expression, leading to increased IL-22 production in the colonic tissues of IBD mice (148). This indicates that CK likely exerts anti-IBD effects through AHR activation and drug-metabolizing CYP enzymes, thus suggesting its action via the gut microbiota-Trp metabolite-AHR axis to mitigate IBD.

### 5.4 Polysaccharides

Natural polysaccharides, derived from diverse sources such as marine organisms, plants, and microorganisms, exhibit broad bioactivities including anti-inflammatory, immunomodulatory, and antioxidant effects (200). They also function as prebiotics by selectively modulating the gut microbiota. These polysaccharides alleviate IBD primarily via the “microbe-ligand-AHR” axis. This mechanism involves regulating gut microbiota to enhance the production of AHR ligands, leading to AHR pathway activation, which in turn upregulates IL-22, strengthens tight junction protein expression, and inhibits inflammatory responses. Furthermore, certain polysaccharides can cooperatively activate protective autophagy or the Nrf2 antioxidant system, thereby promoting intestinal homeostasis through multiple synergistic pathways.

β-Glucan (BG) is a bioactive dietary fiber commonly found in yeast, fungal cells, and certain cereals such as oats, which exerts therapeutic effects against IBD, colorectal cancer, and related diseases primarily through immune modulation, anti-inflammatory actions, antioxidant activity, and microbiota regulation (201). Previous studies have found that oral administration of oat BG and yeast BG can ameliorate DSS-induced colitis in mice, but the mechanisms have not been clarified (202, 203). Recent studies have shown that the mechanism by which yeast BG attenuates DSS-induced colitis is closely related to modulation of the gut microbiota, enhancement of the intestinal barrier function, and inhibition of the IL-17 inflammatory pathway (204). Notably, research by Zhang et al. revealed that *Bacteroides uniformis* degrades BG to produce nicotinamide, which promotes the proliferation of *Lactobacillus johnsonii*. The latter subsequently activates AHR through the generation of ILA, thereby alleviating colitis (149). This discovery underscores the pivotal role of the “microbiota-metabolite-AHR axis” in disease mitigation.

Kiwifruit polysaccharide (KFP), one of the primary active constituents of kiwifruit, exhibits diverse biological activities, including antitumor, antibacterial, antioxidant effects, and intestinal health maintenance (205). Research by Li et al. demonstrated that KFP increases colonic levels of Trp metabolites by modulating the gut microbiota (150). This subsequently activates the AHR/IL-22 pathway, promoting tight junction protein expression and colonic fucosylation, thereby enhancing intestinal barrier function and ameliorating colonic inflammation. Specifically, KFP alleviates UC primarily by targeting gut microbiota involved in the AHR pathway and upregulating colonic fucosylation.

Astragalus polysaccharides (APS), a bioactive compound isolated from the roots of the traditional Chinese herb *Astragalus membranaceus*, exhibit antitumor, antifibrotic, and antioxidant properties (206). While previous studies have elucidated certain pharmacodynamic mechanisms of APS in inflammation, their role in IBD remained unexplored. Based on this, some scholars conducted animal experiments and found that APS improved the intestinal barrier function in inflammatory injury in mice with colitis. Mechanistic research demonstrated that APS triggers AHR activation, where the activated AHR binds to the *Becn1* promoter to promote autophagy, facilitating the transcription of genes involved in anti-inflammatory responses and intestinal barrier repair (151). Thus, APS protects against experimental colitis through an AHR-dependent autophagy mechanism. Furthermore, recent studies also identify APS as a promising prebiotic agent for maintaining intestinal health and confirm its ability to ameliorate colitis in a gut microbiota-dependent manner (207).

Fucoidan, a sulphated polysaccharide derived from brown seaweed, has demonstrated efficacy in ameliorating UC by modulating colonic pathology, cytokine gene expression, and *Enterobacteriaceae* populations (208, 209). Baghagar et al. further confirmed that fucoidan alleviates UC symptoms by upregulating AHR, IL-22, cAMP, Nrf2, and *HO-1* at both transcriptional and protein levels, while concomitantly suppressing phosphodiesterase 4 (PDE4) expression and enhancing antioxidant activity (152). This study provides the first evidence that fucoidan exerts its therapeutic effects via synergistic modulation of the AHR/PDE4 pathway and the Nrf2/ *HO-1*antioxidant system.

Turmeric, a traditional medicinal substrate and food additive, contains core bioactive components including polyphenols and polysaccharide complexes (210). While previous research primarily focused on curcumin's therapeutic effects for IBD (211, 212), novel evidence indicates that turmeric polysaccharide ameliorates DSS-induced UC in mice by remodeling gut microbiota structure—significantly increasing the abundance of probiotic bacteria such as Lactobacillus and *Clostridia-UCG-014*. This remodeling promotes the production of Trp metabolite IAA, thereby activating the AHR signaling pathway. The pathway-mediated upregulation of IL-22 and enhanced tight junction protein expression collectively restore intestinal barrier function, revealing the mechanism by which turmeric polysaccharide modulates intestinal homeostasis through the “microbiota-Trp-AHR axis” (153).

### 5.5 Other special categories

#### 5.5.1 Sugar derivatives

L-fucose is an important natural monosaccharide mainly derived from brown algae and microalgae, with a variety of biological functions including anti-tumor effects and alleviation of intestinal diseases (213). Previous studies have demonstrated that L-fucose administration effectively mitigates DSS-induced murine colitis by reducing intestinal inflammation and restoring the intestinal epithelial barrier (214, 215). Recent studies have shown that L-fucose exhibits therapeutic effects in alleviating DSS-induced chronic colitis in mice by promoting ISC proliferation, and this effect is mediated through the AHR/IL-22 pathway in lamina propria mononuclear cells (LPMCs) (154). These results suggest that L-fucose may be a potential dietary therapy for IBD; however, further studies are necessary to verify its specific therapeutic effects and mechanisms in clinical trials.

Fructooligosaccharides (FOS), naturally occurring oligosaccharides primarily derived from plants such as chicory, asparagus, and onion, function as prebiotics to modulate the gut microbiota and the immune system (216, 217). Studies indicate that FOS may alleviate UC through gut microbiota-dependent Trp metabolism associated with AHR activation, particularly by increasing the colon formation of IAA and IPA and elevating colonic AHR and IL-22 expression levels (155). This suggests that the IAA/IPA-AHR-IL22 axis may mediate the anti-colitis effects of FOS.

#### 5.5.2 Polyamines

Spermidine (SPD) is a widely occurring natural polyamine that mediates the self-repair function of the intestinal mucosa and treats colitis by regulating the gut microbiota and immune cells (218, 219). Research indicates that SPD interacts with the polycationic binding site of AHR, participating in stabilizing the active conformation of AHR bound to DNA and promoting the non-specific binding of the receptor to DNA. Its cellular concentration may influence AHR activity (220). Studies in IBD models demonstrate that SPD alleviates intestinal barrier defects by reducing hyperpermeability, restoring transepithelial electrical resistance, and normalizing tight junction distribution, thereby inhibiting bacterial translocation. This protective effect is mediated through AHR activation, as SPD upregulates AHR expression at both mRNA and protein levels in mouse colon tissues and Caco-2 cells, while inducing its downstream target *Cyp1a1*. AHR activation by SPD triggers a dual pathway response, promoting Nrf2 expression and nuclear translocation to drive antioxidant gene expression, while concurrently inhibiting *STAT3* phosphorylation to suppress pro-inflammatory signaling. Collectively, SPD enhances intestinal barrier function in colitis through the AHR-Nrf2 and AHR-*STAT3* pathways, suggesting a potential therapeutic strategy for IBD (156).

#### 5.5.3 Natural extract

Indigo Naturalis (IN), a traditional Chinese medicine characterized by its cold property and salty taste, is historically used to clear heat and detoxify, cool blood, resolve maculae, and purge fire. It is derived from processed dried powders or masses of *Strobilanthes cusia*, *Polygonum tinctorium*, or *Isatis tinctoria* (221). Its biological activity primarily stems from its diverse array of compounds, with key constituents including indole and quinazolinone alkaloids (such as indigo, indirubin, and tryptanthrin), as well as critical functional ligands like I3A. Modern pharmacological studies reveal that, beyond its traditional applications, IN exhibits anti-inflammatory, antibacterial, antiviral, analgesic, hemostatic, immunomodulatory, anticancer, antitumor, and antileukemic activities (222). Research demonstrates that IN and indigo administration ameliorate DSS-induced murine colitis by activating the AHR pathway, likely through AHR-mediated upregulation of IL-10 and IL-22 (91). Furthermore, it promotes ulcer healing in UC by activating the AHR and enhancing the proliferation and migration of colonic epithelial cells (157).

*Schisandra chinensis*, known for its astringent and consolidating properties, qi-tonifying effects, fluid-promoting actions, and kidney-nourishing and mind-calming functions, is commonly used to treat UC due to its antidiarrheal activity (223, 224). Modern research has confirmed that its efficacy is primarily derived from core components such as lignans, polysaccharides, volatile oils, and others. In the DSS-induced chronic murine UC model, vinegar-processed *Schisandra chinensis* improves colonic inflammation and comorbid depressive-like behavior by regulating gut microbiota, reprogramming Trp metabolism, activating the AHR pathway, and inhibiting the NF-κB inflammatory pathway, thereby repairing the intestinal mucosa and the blood-brain barrier (158).

### 5.6 Microbial metabolic products

The gut microbiota functions as a critical “biological factory” responsible for the endogenous production of AHR ligands. Among its metabolic products, Trp-derived metabolites are particularly crucial in maintaining gut homeostasis and intervening in IBD. Microbial-derived AHR ligands are attractive therapeutic molecules due to their endogenous origin, tissue specificity, and relatively low toxicity profile (225). Although research on microbiota-derived metabolites is still in its early stages, progress has been made in studying how they modulate the AHR signaling pathway to intervene in IBD. This section focuses on elucidating the mechanisms by which microbiota-derived metabolites with potential therapeutic value intervene in IBD through the regulation of AHR. (**Table 3**).

**Table 3:** Microbe-derived metabolites with potential to treat IBD via AHR.

Microbial products	Source	Producer microorganisms	Mechanism	Ref.
ILA	Trp	*Lactobacillus plantarum DPUL-S164*, *Bifidobacterium bifidum*, *Bifidobacterium longum subsp. Infantis*	Activated AHR to upregulate TJs (e.g., ZO-1, Claudin-1) and protect the integrity of the intestinal barrier; Activated the AHR-Nrf2 pathway	(108, 226)
IAA	Trp	*Lactobacillus reuteri*, Bacteroides spp.	Activated the AHR-Papss2-Slc35b3 pathway	(40)
IPA	Trp	*Clostridium* sp*orogenes*	Activated AHR-IL22 axis; Activated AHR to induce IL-10R1 expression and improve intestinal barrier formation	(27, 155)
IAld	Trp	*Lactobacillus reuteri*	Activated AHR to inhibit ROS production and NLRP3 inflammasome	(227)
IA	Trp	*Clostridium paraputrificum Clostridium* sp*orogenes*	Activated AHR to improve intestinal barrier function and immune regulation	(228)
UroA	Foods rich in ellagitannins (pomegranates, berries, walnuts)	*Gordonibacter pamelaeae Gordonibacter urolithinfaciens Eubacterium ramulus*	Activated the AHR-Nrf2 pathway	(37, 229)
phloroglucinol	dietary polyphenols	*Clostridium perfringens Clostridium butyricum Bacteroides thetaiotaomicron*	AHR-macrophage inflammation	(230)

Empirical evidence demonstrates that ILA, metabolized by *Lactobacillus plantarum DPUL-S164*, acts as an endogenous AHR ligand that alleviates DSS-induced disruption of the intestinal epithelial barrier and attenuates gut inflammation in UC murine models. This protective effect is mediated through the coordinated activation of the AHR-Nrf2 pathway alongside inhibition of NF-κB signaling (226). Similarly, ILA synthesized by *Bifidobacterium bifidum* strains FL-276.1 and FL-228.1 enhances epithelial barrier integrity and reduces inflammation through modulation of the AHR/Nrf2/NLRP3 signaling axis (108). Notably, IAA produced by *Lactobacillus reuteri* activates the AHR-Papss2-Slc35b3 pathway, promoting intestinal mucin sulfation, thereby maintaining gut homeostasis and alleviating colitis. The loss of this protective effect in an IAA synthesis-deficient strain substantiates the IAA-AHR axis as a viable therapeutic target for IBD (40). IPA, a principal Trp metabolite primarily generated by *Clostridium* sp*orogenes*, exerts anti-inflammatory effects in mice through activating AHR signaling (27, 155). IAld similarly activates AHR, suppresses ROS production, and blocks the NF-κB/NLRP3 inflammatory pathway within IECs, thereby preserving barrier function and highlighting its therapeutic potential (227). IA, another potent microbiota-derived Trp metabolite, exhibits marked anti-inflammatory activity by enhancing intestinal barrier function and immune regulation through upregulation of MUC2 expression and AHR activation, while concurrently alleviating anxiety-like behaviors (228). This dual physical and psychological therapeutic effect presents a novel strategy for IBD. Urolithin A (UroA), a gut metabolite derived from ellagitannin-rich foods, exerts anti-inflammatory, antioxidant, and anti-aging effects. It enhances intestinal barrier function by directly activating AHR and engaging the AHR-Nrf2 synergistic pathway, which upregulates the expression of TJs and key metabolic enzymes (37, 229). Additionally, Phloroglucinol, a key microbial-derived end product of dietary polyphenol degradation, is clinically employed in the treatment of irritable bowel syndrome (IBS) owing to its spasmolytic activity and has shown anti-inflammatory potential *in vitro*. Notably, recent research indicates that phloroglucinol alleviates intestinal inflammation by targeting the AHR pathway. Phloroglucinol activate AHR pathway to target hematopoietic stem/progenitor cells, thereby promoting an anti-inflammatory macrophage phenotype and central trained immunity, a process dependent on AHR-cofactor coordination and sustained regulation of downstream transcriptional and metabolic programs (230).

Although AHR-targeting microbe-derived metabolites have demonstrated potential for treating IBD in preclinical studies, their further development faces several limitations. Similar to many natural products, these metabolites may share common pharmacokinetic drawbacks, including poor ligand stability, low oral bioavailability, and short intestinal residence time, which constrain the full realization of their therapeutic potential. In addition, long-term safety and the potential risk of microbial imbalance remain to be fully assessed, and there are technical challenges in large-scale preparation and efficient delivery (231). Given AHR's central role in maintaining intestinal immune homeostasis, addressing these obstacles is imperative. Future research should prioritize exploring novel strategies, such as leveraging engineered probiotics for *in situ* colonization and metabolite delivery, or developing structurally stabilized analogs derived from key metabolite frameworks.

## 6 Conclusions, challenges, and future directions

This paper systematically elucidates the critical role of AHR as a central hub integrating environmental signals and intestinal homeostasis in the pathogenesis of IBD. Specifically, the AHR signaling pathway has been demonstrated to regulate IBD progression through three core mechanisms. First, it maintains the integrity of the intestinal epithelial barrier, including controlling the function of IECs, modulating the expression of TJs, mucus layer synthesis, and antimicrobial peptides secretion. Second, it balances the differentiation of immune cell subpopulations and remodels the cytokine network. Third, it facilitates microbiota-host interactions by integrating Trp metabolite signals through the “microbe-ligand-AHR” axis to coordinate immune response and barrier functions. Numerous studies have demonstrated that natural compounds from diverse structural classes, including microbial-derived metabolites, can significantly alleviate intestinal inflammation, barrier damage, and microbiota dysbiosis in IBD animal models by targeting the AHR pathway, which confirms the value of AHR as a potential target for therapeutic intervention in IBD. Among these, microbial-derived Trp metabolites, acting as endogenous AHR ligands, directly activate AHR or synergize with dietary components, offering novel approaches for precision interventions in IBD. More importantly, natural compounds, due to their low toxicity, multi-component synergistic effects, and ability to regulate the microbiome, can effectively avoid the potential carcinogenic risks associated with synthetic AHR agonists, offering a more promising intervention strategy for the long-term safe treatment of IBD.

However, significant limitations and challenges remain in current research. First, the key evidence for AHR in IBD originates from animal models, and its cell-specific expression and the dynamic mechanisms of its signaling pathways in human intestinal tissues have not yet been clarified (225). Second, although various natural products and microbial metabolites have been confirmed to exert therapeutic effects via AHR, the specific type of AHR signaling pathway (canonical or non-canonical) underlying their actions has not been clearly distinguished or validated in most studies. Additionally, beyond established ligands, many natural products with AHR-modulatory activity have been characterized only functionally, and their direct receptor-binding mechanisms require validation through standardized experimental approaches (232). Third, there is a “duality” of AHR ligands: low-concentration activation exerts anti-inflammatory protection, while high-concentration environmental pollutants induce toxicity. This is not only related to the activation concentration of AHR but also closely associated with ligand-specific effects. For instance, the effective doses of natural AHR agonists in rodent IBD models, summarized in this review (e.g., Quercetin, 20-50 mg/kg; Baicalein, 10-40 mg/kg), are often achieved through pharmacological supplementation and generally exceed concentrations attainable from diet alone. This disparity underscores the need to establish a therapeutic window that balances efficacy and safety, distinct from the toxicity associated with high-affinity environmental ligands. Furthermore, ligand-specific effects arise because different ligands induce distinct conformational changes in the AHR ligand−binding domain. These alterations can modulate AHR affinity, nuclear translocation, complex stability with ARNT, and ultimately the recruitment of specific transcriptional co−regulators (18). Fourth, substantial inter−individual variation in microbiota composition and metabolic networks leads to significant differences in endogenous AHR ligand production, directly affecting the reproducibility and personalized application of intervention strategies. Furthermore, the source type of natural products, whether dietary or medicinal, critically influences their research and translation strategies. Dietary AHR ligands (e.g., polyphenols from fruits and vegetables) benefit from an established safety profile through long-term consumption and the practicality of nutritional intervention (233). Research on them focuses on defining effective dietary doses, improving bioavailability, and developing functional food applications. In contrast, medicinal active ingredients (e.g., many alkaloids and terpenoids) typically possess higher pharmacological potency. Their investigation prioritizes determining therapeutic windows, mitigating potential toxicity, and developing drug-appropriate delivery systems. Finally, the application of natural compounds faces multiple challenges: their therapeutic efficacy, often mediated through the AHR pathway, is difficult to attribute due to multi-component interactions conclusively; most natural compounds suffer from low bioavailability, significant first-pass metabolism, and a lack of efficient targeted delivery systems; and the specific structural basis and synergistic mechanisms underlying the interactions between active components in complex systems and AHR remain unclear.

Future research should focus on the following directions. At the mechanistic level, it is necessary to analyze the complex regulatory network of the AHR pathway deeply. Utilizing human intestinal organoids in conjunction with single-cell multi-omics technology, researchers should map the cell-type-specific AHR signaling profiles across diverse immune and epithelial cell subsets (234). Particular attention should be paid to species differences in AHR ligand responses and microbiota metabolic pathways between rodents and humans (235). The use of human microbiota-colonized mouse models should be prioritized to evaluate the regulatory effects of human-specific metabolites. At the technical level, it is crucial to optimize the design of AHR ligands and their delivery efficiency. On the one hand, novel AHR agonists with high selectivity and low toxicity should be designed and screened based on the structural features of the AHR ligand-binding domain, aiming to strike a balance between their therapeutic potential and safety. On the other hand, advanced delivery systems-such as pH-responsive PLGA microparticles (236) and plant exosome-like nanoparticles (237)-should be developed to overcome the limitations of natural compounds. These systems would enable the targeted release of medications at colonic inflammatory sites, thereby enhancing bioavailability and efficacy. Regarding microbiota modulation, precise regulation of the “microbiota-AHR axis” is necessary. To this end, functional bacterial strains that can efficiently convert Trp into highly active AHR ligands should be screened and identified. A triple therapy based on “probiotics-prebiotics-AHR ligands” should be developed. Concurrently, personalized prediction models for AHR ligand levels should be established by combining metabolomics and macro-genomics, guiding precise dietary and microbiological interventions. Finally, clinical trials utilizing natural AHR ligands and optimized delivery systems should be conducted to evaluate their efficacy and safety in repairing intestinal barriers, regulating immunity, and restoring microbiome homeostasis in IBD patients, thereby providing patients with more effective treatment options.

### 6.1 Search methodology

To elucidate the role of AHR in IBD and its potential as a therapeutic target for natural products, this review collected relevant literature up to September 2025. The search focused on three areas: AHR structure and signaling pathways (since 1994), its regulatory role in IBD pathogenesis (with emphasis on the last decade), and natural products targeting AHR, including phenolic compounds, alkaloids, terpenoids, polysaccharides, and microbial metabolites. Databases such as PubMed, Web of Science, Scopus, and ScienceDirect were used, with keywords including “aryl hydrocarbon receptor”, “AHR”, “inflammatory bowel disease”, “IBD”, “natural products”, “intestinal barrier”, “gut microbiota”, and related terms. Searches were conducted independently by two researchers, followed by cross-checking and a full-text review to ensure the comprehensive and accurate inclusion of literature.
